# Host-pathogen biotic interactions shaped vitamin K metabolism in Archaeplastida

**DOI:** 10.1038/s41598-018-33663-w

**Published:** 2018-10-15

**Authors:** U. Cenci, H. Qiu, T. Pillonel, P. Cardol, C. Remacle, C. Colleoni, D. Kadouche, M. Chabi, G. Greub, D. Bhattacharya, S. G. Ball

**Affiliations:** 1 Unité de Glycobiologie Structurale et Fonctionnelle, UMR 8576 CNRS-USTL, Université des Sciences et Technologies de Lille, Bâtiment C9, Cité Scientifique, 59655 Villeneuve d’Ascq Cedex, France; 2 Department of Ecology, Evolution & Natural Resources, Rutgers University, New Brunswick, NJ 08901 USA; 3 Center for Research on Intracellular Bacteria (CRIB), Institute of Microbiology, University Hospital Center and University of Lausanne, 1011 Lausanne, Switzerland; 4 Laboratoire de Génétique et Physiologie des Microalgues, InBioS/Phytosystems, B22 Institut de Botanique, Université de Liège, 4000 Liège, Belgium; 5 Department of Biochemistry and Microbiology, Rutgers University, New Brunswick, NJ 08901 USA

## Abstract

Menaquinone (vitamin K_2_) shuttles electrons between membrane-bound respiratory complexes under microaerophilic conditions. In photosynthetic eukaryotes and cyanobacteria, phylloquinone (vitamin K_1_) participates in photosystem I function. Here we elucidate the evolutionary history of vitamin K metabolism in algae and plants. We show that Chlamydiales intracellular pathogens made major genetic contributions to the synthesis of the naphthoyl ring core and the isoprenoid side-chain of these quinones. Production of the core in extremophilic red algae is under control of a menaquinone (Men) gene cluster consisting of 7 genes that putatively originated via lateral gene transfer (LGT) from a chlamydial donor to the plastid genome. In other green and red algae, functionally related nuclear genes also originated via LGT from a non-cyanobacterial, albeit unidentified source. In addition, we show that 3–4 of the 9 required steps for synthesis of the isoprenoid side chains are under control of genes of chlamydial origin. These results are discussed in the light of the hypoxic response experienced by the cyanobacterial endosymbiont when it gained access to the eukaryotic cytosol.

## Introduction

**Figure 1:**
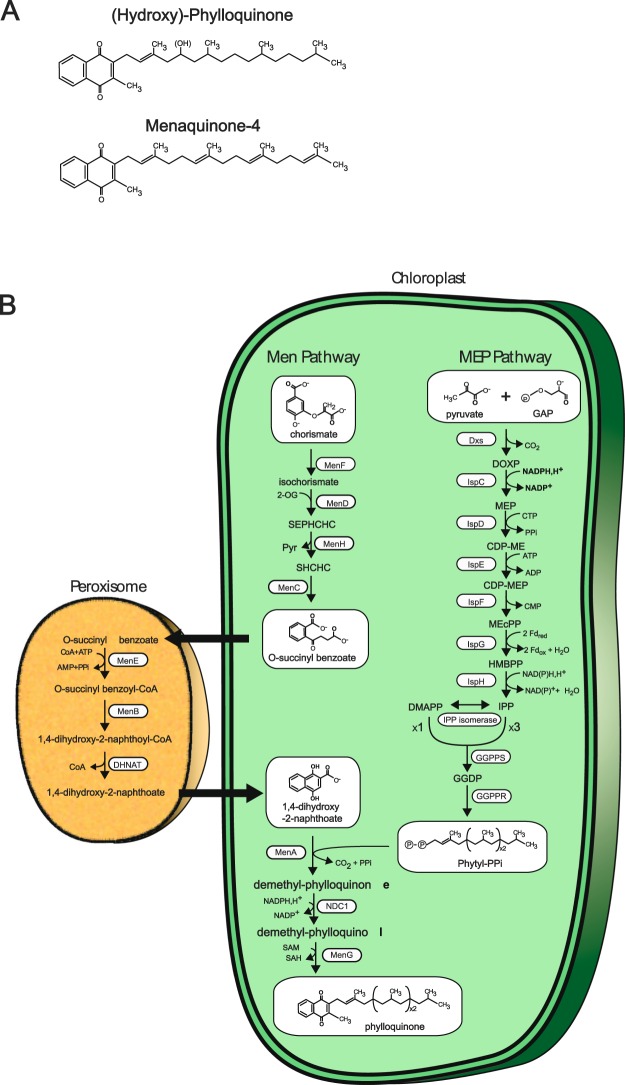
Structures of Vitamin K and biosynthesis in green plastids. (**A**) Chemical structure of Vitamin K_1_ (Phylloquinone) and Vitamin K2 (menaquinone/MK_4_). Phylloquinone is present as a bound co-factor in photosystem 1 (PS1) of most cyanobacteria and plants. Hydroxyphylloquinone has been shown to replace phylloquinone in PS1 of the green alga *Chlamydomonas reinhardtii* and is reported in this work as the major naphtoquinone of the glaucophyte *Cyanophora paradoxa*. Vitamin Ks are composed of a 2-methyl-1,4-naphthoquinone ring coupled to various types of polyprenyl chains. This chain consists of phytyl in phylloquinones which is a reduced form of the geranyl geranyl diphosphate found in menaquinone-4 (MK 4). Menaquinones are characterized by the presence of polyunsaturated polyprenyl chains of various lengths synthesized by polymerization of the MEP pathway 5 carbon products IPP and DMAPP in bacteria. MK4 is characterized by a degree of polymerization of 4 and thus contains 20 carbon units. Menaquinones shuttle electrons between different respiratory complexes in anaerobic respiration or aerobic respiration in a microaerophilic environment. Phylloquinone is unable to supply this particular function. Mutants defective for vitamin K synthesis in algae and cyanobacteria substitute phylloquinone/menaquinone by plastoquinone for PSI function and thereby become highly light sensitive. (**B**) Synthesis of phylloquinone in plants. The end product of the shikimate pathway (chorismate) is converted in 4 steps in the chloroplast to O-succinyl benzoate (OSB) by a large protein called “phyllo” consisting of a fusion of the MenF, MenD, MenC and MenH gene products derived from the bacterial Men pathway of menaquinone synthesis. OSB diffuses out to the peroxisome where it is converted to DHNA (1.4-dihydroxy-2-naphthoate) in 3 steps (catalyzed by MenE, MenB and DHNAT) and diffuses back to the plastid. The isoprenoid polyprenyl (phytyl) tail is added to DHNA by the MenA gene product. The DHNA ring is then reduced by the NDC1 NADPH dehydrogenase and methylated by the MenG gene product. In bacteria MK4 is synthesized by transfer to the quinone core of geranylgeranyl diphosphate (GGPP). Synthesis of GGPP in bacteria and plants involves the transfer of 3 IPP molecules onto a single DMAPP acceptor by geranylgeranyl diphosphate synthase (GGPPS). Phylloquinone instead of MK4 is synthesized specifically in cyanobacteria and plants because geranylgeranyl diphosphate is reduced by GGPP reductase (GGPPR) prior to its transfer to the quinone core by the Men A prenyl transferase. IPP and DMAP are the end products of the 8 steps MEP pathway in cyanobacteria and Archaeplastida.

Membrane-bound polyunsaturated isoprenoid quinones (Fig. 1) undergo a two-step reversible reduction, making them ideal electron shuttles between different protein complexes, such as those involved in respiration and photosynthesis^1^. Ubiquinone and plastoquinones are the predominant electron carriers under aerobic conditions, whereas menaquinones (vitamin K_2_) play a similar role under low oxygen tension. These observations apply both to anaerobic respiration in bacteria^2^ and oxidative phosphorylation under microaerophilic conditions in bacteria and metazoans^2,3^. Reduction of the polyunsaturated isoprenoid side chain of menaquinone, from geranylgeranyl diphosphate to phytyl pyrophosphate, yields after transfer the partially saturated phylloquinone (vitamin K_1_) present in cyanobacteria and most photosynthetic eukaryotes^4^. The presence of fewer double bonds affects phylloquinone membrane diffusion properties, apparently precluding the electron shuttle function. Nevertheless, phylloquinone is used in many cases as a bound co-factor of photosystem I (PSI)^1,4^. Eukaryotes also use menaquinones for various functions. In animals, they are necessary co-factors of blood clotting reactions^4^, but also function as electron carriers in mitochondrial respiration, or lipid metabolism under microaerophilic conditions^3,5^. Animals and most protists do not contain the genes encoding enzymes of menaquinone or phylloquinone synthesis, yet these molecules identified as vitamins are required for survival. External provision of the widespread phylloquinone form of vitamin K requires substitution of the partially saturated isoprenoid phytyl side chain with polyunsaturated prenyl chains. This reaction is carried out by UBIAD1, an essential prenyltranferase derived from the same family (*MenA*) as the bacterial gene responsible for the transfer of the isoprenoid polyunsaturated chain to the quinone core^3^.

Two distinct pathways (Men and Futalosine) are responsible for the conversion of chorismate to menaquinone or phylloquinone in Bacteria and Archaea^6^. Cyanobacteria and plants contain the classical Men pathway illustrated in Fig. 1. In plants, the first four steps, starting from chorismate and ending with o-succinyl benzoate are catalyzed in the plastid by a large nuclear-encoded protein fusion referred to as “PHYLLO” that produces o-succinyl benzoate, used in peroxisomes to yield the DHNA (1,4-dihydroxy-2-naphthoic acid) core of the quinone^4^. This core is reimported into plastids, where it is prenylated by MenA, reduced by Ndc1 (NAD(P)H dehydrogenase C), and methylated by MenG prior to assembly into PSI^4,7^. Previous investigations of the evolutionary history of Archaeplastida (red, green, and glaucophyte algae and plants) Men enzymes revealed them to be of unclear, albeit non-cyanobacterial (i.e., not plastid endosymbiont) origin for a majority of the steps^8^. This includes a Men gene cluster in the plastid genome of the anciently diverged, extremophilic, unicellular red algae, Cyanidiophytina^8,9^. Using recently generated genome data that includes taxonomically diverse environmental Chlamydiales (i.e., Rhabdochlamydiaceae and other deep branching chlamydial taxa that are sisters to both Simkaniaceae and Rhabdochlamydiaceae), we elucidated the phylogenetic history of these key enzymes. Our results provide a biochemistry and genomics-enabled evolutionary perspective on the origin of menaquinone/phylloquinone metabolism in Archaeplastida. These data suggest that obligate intracellular bacterial pathogens of the order Chlamydiales may have interacted directly with the cyanobacterial ancestor of plastids through lateral gene transfers (LGTs) to the ancestral plastid genome. The timing and implications of these transfers are discussed in our paper.

## Results

### Origin of Individual Men genes contained in Cyanidiophytina

Most Cyanidiophytina inhabit aqueous hot springs environments, and with the exception of *Galdieria sulphuraria* and *Galdieria phlegrea*, a plastid gene cluster encoding the *MenF*, *D*, *C*, *E*, *B*, and *MenA* genes^8^. Our analysis of the cluster provides evidence that the gene encoding (DHNA)-CoA thioesterase (*ycf83*) is also physically linked to the Men genes in Cyanidiophytina. This expanded cluster defines a complete menaquinone synthesis pathway up to DHNA, with the exception of *MenH* that catalyzes the third step in the synthesis of the quinone core and is a component of the plant nuclear and mesophyllic rhodophycean phyllo fusions^4^ (this work). *MenH* is however often absent from bacterial genomes^8^ that encode the classical Men pathway, suggesting conditional requirement for this catalyst based on metabolite concentrations. At variance with all other red algae, Cyadiniophytina lack PHYLLO-like nuclear gene fusions.

**Figure 2:**
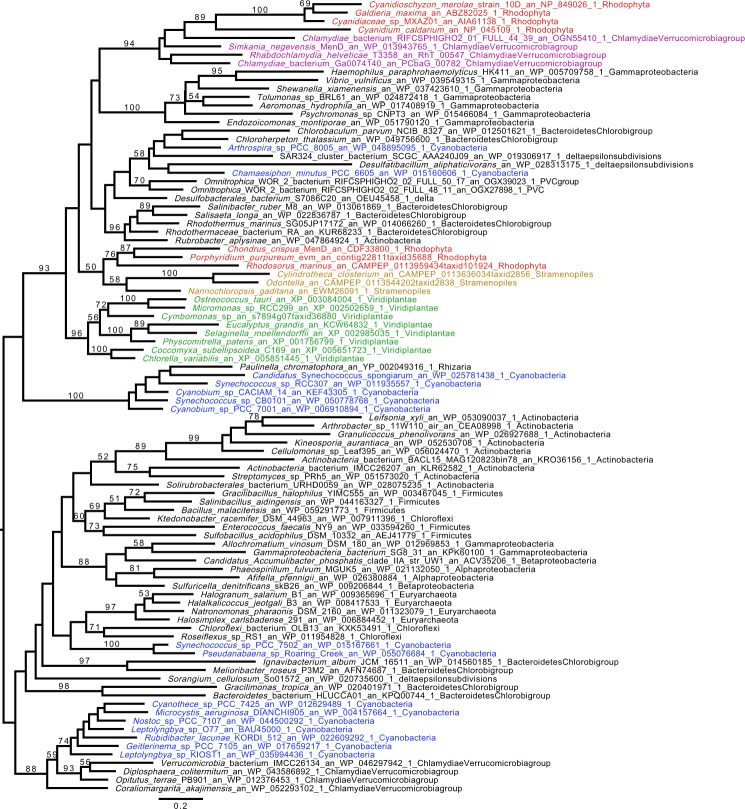
Phylogenetic analysis of the plastid Cyanidiophytina MenD genes. The tree shown was obtained with IQ-TREE (Nguyen *et al*. 2015) obtained with the C20 model, bootstrap values are displayed onto the nodes. Bootstrap values (BV) >50% are shown. The tree is midpoint rooted. Sequences are colored according to their taxonomic affiliation: Chlamydiae (purple), Cyanobacteria (dark blue), Viridiplantae (green), Rhodophyta (red), and algae bearing a plastid obtained by secondary or tertiary plastid endosymbiosis (brown). Other organisms are in black. The scale bar shows the inferred number of amino acid substitutions per site. MenD from the Cyanidiophytina and Chlamydiae show a common origin with BV of 94.

**Figure 3:**
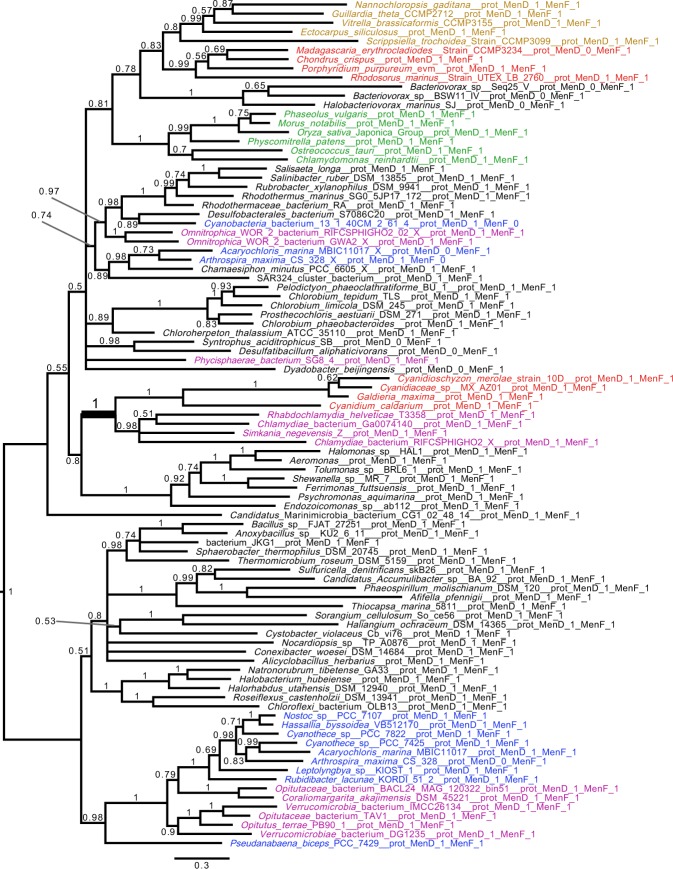
Concatenated phylogenetic analysis of MenFD (Bayesian analysis). Phylogenetic trees of the concatenated MenF and MenD proteins with Chlamydiales. The tree was inferred with phylobayes^46^ under the CAT + GTR model^47^. We used stringent criteria and considered convergence when maxdiff <0.1 were obtained based on sample trees (taking one from every 100 trees) after a minimum of at least 300 trees. We assessed the burn-in to be 320. The tree is midpoint rooted. The scale bar shows the inferred number of amino acid substitutions per site. The Chlamydiales are in purple, Rhodophyta are in red, Chloroplastida are green, algae bearing a plastid obtained by secondary or tertiary plastid endosymbiosis are brown, all other taxa are displayed in black. The tree shows a clear relationship between Cyanidiophytina and Chlamydiales with a posterior probability = 1. Note that MenD from *Bacteriovorax* is distantly related to the archaeplastidal sequences and only MenF shows this affiliation.

**Figure 4:**
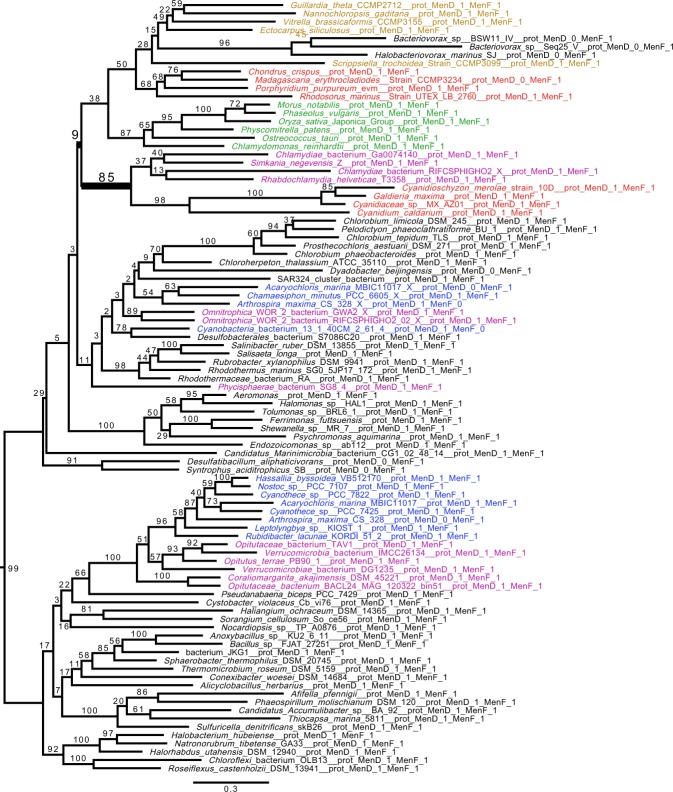
Concatenated phylogenetic analysis of MenFD (ML analysis). The tree is performed using IQ-TREE^48^ under the C30 model^49^. The tree was rooted manually, to facilitate comparison with the Bayesian tree (Fig. 3). The scale bar shows the inferred number of amino acid substitutions per site. Color codes are the same as in Fig. 3. The tree shows a clear relationship between Cyanidiophytina and Chlamydiales with bootstrap support = 85. The C30 model shows that Archaeplastida are monophyletic even if the statistical support values are very low for MenD and MenF and have a chlamydial origin. Note that MenD from *Bacteriovorax* is distantly related to the archaeplastidal sequences and only MenF shows this affiliation.

We inferred phylogenetic trees for all enzymes involved in the synthesis of the menaquinone core structure (maximum likelihood [ML] analysis under the C20 or C10 models and Bayesian analysis under the CAT + GTR model). We show a robust affiliation of Cyanidiophytina *MenD* to Chlamydiales (bootstrap value [BV] = 94% for ML [C20 model] analysis; Bayesian posterior probability [BP] = 1) (Fig. 2). The MenF tree showed a comparable topology with the same Simkaniaceae and Rhabdochlamydiaceae clades grouping with the plastid cluster, however with poor statistical support (Supplementary Fig. S1). These results suggest a common evolutionary history for these genes. ML analysis under the C20 model produced topologies for both proteins in which the Cyanidiophytina were nested within the Chlamydiales suggesting LGT from the latter to the red algal plastid genome (Fig. 2 and Supplementary Fig. S1). However, the interrelationship of Chlamydiales was poorly resolved in all these trees and the Bayesian analysis displayed an alternative, poorly supported sister group relationship between them (Supplementary Figs S2 and S3). The phylogenetic results are unresolved for other members of this cluster (*MenA, MenB, MenC, MenE, DHNAT*), but all of these trees argue against a cyanobacterial provenance of the genes (Supplementary Figs S4–S8). Despite the lack of resolution in all single gene trees, proteins in the Cyanidiophytina plastid cluster form a monophyletic group, suggesting a common origin for all genes of the plastid cluster. It is likely that extensive gene sharing among bacteria, and the paucity of informative sites on these small proteins, explain this lack of phylogenetic resolution. To aid phylogenetic resolution, we concatenated the MenF and MenD alignments after checking their congruence. The node uniting MenFD of Cyanidiophytina with Chlamydiales to the exclusion of all other bacteria was well supported (BP = 1; BV = 85% for ML [C30 model]) with this analysis, (Figs 3 and 4). The concatenated Bayesian analysis suggested a sister group, rather than a nested relationship between Cyanidiophytina and Chlamydiales but this alternative topology also received weak support (BV = 37%). Our results are thus compatible with two distinct scenarios: both Chlamydiales and Cyanidiophytina gained the MenFD encoding genes independently from an as-yet unidentified bacterial source (consistent with the sister group relationship suggested by the Bayesian analysis) or, alternatively, the Cyanidiophytina gained these genes from a chlamydial source (consistent with the nested ML-derived topology). We reasoned that if these sequences were acquired from a bacterial source other than Chlamydiales, then deleting Chlamydiales from the alignment should strengthen the relationship with these bacteria, given that related genomes from these donors are in our dataset (i.e., we cannot account for missing data). Surprisingly, this deletion increased both bootstrap and Bayesian support for Archaeplastida monophyly and attracted the long-branched Cyanidiophytina to this supergroup (BV = 47; BP = 0.92; Supplementary Figs S9 and S10), arguing against a non-chlamydial source for Cyanidiophytina MenFD. We do not favor the scenario that the Cyanidiophytina plastid sequences were donors of MenFD to other bacteria because these sequences are not of cyanobacterial provenance and are, in fact, absent from the cyanobacterial clade most closely affiliated with plastids (see below).

We interpret these phylogenetic data as follows: (1) the monophyly of Cyanidiophytina in all Men protein trees indicates single, ancient origins of the genes in the plastid genome of the common ancestor of this red algal clade, and (2) the physical association of the plastid-encoded genes means they likely occurred as a cluster in this ancestral lineage, with no subsequent LGTs (i.e., gene transfer into plastid DNA is exceedingly rare). (3) The absence of all Men genes with the exception of MenA (UBIAD1) in eukaryotes unrelated to Archaeplastida argues that the transfer was from a bacterial source. Given these assertions are correct, we suggest that the intact Men cluster entered the plastid genome of the Cyanidiophytina ancestor from a chlamydial source and has been maintained since its origin. Some of the Men genes however may have had a chimeric (i.e., non-chlamydial) origin in the donor cluster prior to plastid endosymbiosis.

### Origin of the “phyllo” gene fusion

The nuclear encoded Archaeplastida “phyllo” fusion proteins and the corresponding plastid cluster Men enzymes from Cyanidiophytina are polyphyletic in the single gene trees, as well as in the MenFD Bayesian analysis (Fig. 3, Supplementary Figs S6 and S11). ML analysis of MenFD provides evidence for Archaeplastida monophyly (Fig. 4), inclusive of Cyanidiophytina, but this topology has no bootstrap support (BV = 9%). A previous analysis reported the monophyly of MenFD phyllo for all red and green algae inclusive of the Cyanidiophytina^8^. We do not recover this topology in our concatenated analysis unless we delete Chlamydiales from the alignment (Supplementary Fig. S9). However, even in this case, statistical support for this result is weak or at best moderate. Therefore, we cannot presently discriminate between a single early transfer and two independent transfers of Men genes encoding the phyllo fusion and the Cyanidiophytina plastid cluster. Note that the “phyllo” fusions also contain the MenC (Supplementary Fig. S6) and MenH (Supplementary Fig. S11) proteins but unfortunately these phylogenies are unresolved.

### Nuclear Men genes involved in downstream processes in Archaeplastida

Inference of the phylogeny of nuclear-encoded Men genes whose products act downstream from DHNA (thus excluding phyllo and the peroxisomal enzymes) in Archaeplastida provides a complex set of results. For *MenA*, some prasinophyte green algae contain a gene encoding a second isoform shared with Chlamydiae and a restricted number of deep branching PVC (Planctomycete-Verrucomicrobia-Chlamydia supergroup) (BP = 1, BV = 92%) (Supplementary Fig. S4). This is in addition to the cyanobacterial homolog (Supplementary Fig. S12) that is present in all green algae and mesophilic Rhodophyta. As is often the case in these “deep time” phylogenies, directionality of gene transfer is unresolved. However, the restricted distribution of this isoform to a few prasinophytes among eukaryotes suggests that the Chlamydiae are much more likely to be donors than recipients in these phylogenies. The additional chlamydial-derived MenA in prasinophytes contains a eukaryotic signal sequence, a feature discriminating it from cyanobacterium-derived plastidial prenyltransferase isoforms that contain a plastid targeting sequence. UBIAD1, a MenA-like prenyltransferase that is widely distributed among eukaryotes also contains a signal peptide predicting an ER localization but is reported to partition between eukaryotic endomembranes and mitochondria for respiration in microaerophilic environments and in lipid metabolism^3,5^. Presence of cyanobacterial MenA prenyltransferase in Archaeplastida (Supplementary Fig. S12), with the exception of Cyanidiophytina, indicates the requirement for this protein for assembly of quinone onto PSI, as has previously been reported for cyanobacteria and green algae. This requirement was apparently lost in the Cyanidiophytina ancestor, leading to the association of menaquinone rather than phylloquinone with PSI^10^. This occurred with either the involvement of a nuclear UBIAD1 transferase (*G. sulphuraria*) (Supplementary Fig. S12) or of the plastid gene (Supplementary Fig. S4).

In the case of *MenG*, all red algae contain a bacterial gene, possibly of PVC origin (Supplementary Fig. S13A) ((BP = 0.99 for the node uniting red algae with Verrucomicrobia + *Thermotoga maritima*, BV = 37% for positioning Verrucomicrobia as proximal to Archaeplastida), whereas glaucophytes and green algae plus plants contain the cyanobacterium derived homolog, presumably via EGT from the plastid genome (Supplementary Fig. S13B). Finally, *Ndc1* encoding the NADPH oxidase responsible for reduction of the prenylated quinone that is required for its methylation by the MenG methylase is of cyanobacterial (i.e., endosymbiotic) provenance in all Archaeplastida (Supplementary Fig. S14).

### Origin of glaucophyte Men genes

**Figure 5:**
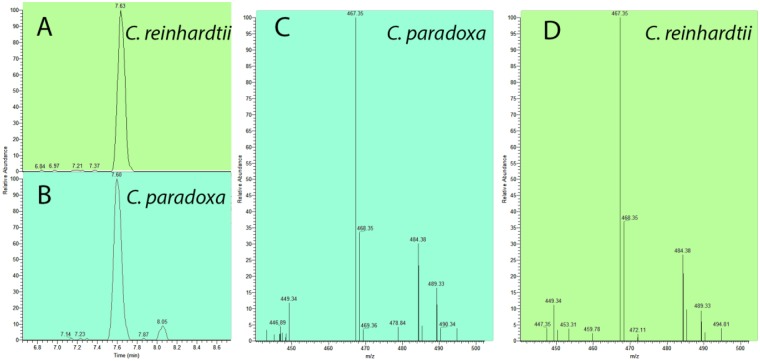
Characterization of glaucophyte naphtoquinone content. Identification of monohydroxylphylloquinone in *Chlamydomonas reinhardtii* and *Cyanophora paradoxa* by ultra-performance liquid-chromatography (UPLC, **A,B**) (left panel: (**A**) *Chlamydomonas reinhardtii* and (**B**) *Cyanophora paradoxa* extracts) followed (**C** and **D**) by mass spectrometry (MS, **C**,**D**). monohydroxylphylloquinone whose determined m/z values are 467.35 and 489.33 for the non-adduct form and Na+ adduct form, respectively, was detected as a single peak at ~7.6 min of the chromatogram (selected monitoring mode, m/z = 466.5–467.5).

An unexpected finding of our study was the absence of the classical Men pathway upstream of DHNA in both glaucophytes and *G. sulphuraria*. An important exception is *MenB* that was identified in the *Cyanoptyche gloeocystis* transcriptome assembly (Supplementary Fig. S5). We do not believe this result is explained by missing data, because we located *MenA* and *MenG* in all glaucophytes and in *G. sulphuraria*. Because we were unable to find a published account of the quinone content of glaucophyte PSI, we searched for evidence of naphtoquinones in *Cyanophora paradoxa* cell extracts. We report the presence of hydroxyphylloquinone in these algae (Fig. 5). It is notable that several phylogenomic studies have placed the ancestor of plastids among the most basal cyanobacterial clades^11,12^. A recent report identified the Gloeobacterales *Gloeomargarita lithophora*^11^ as the closest extant relative of the plastid donor. A previous study demonstrated the presence of menaquinone rather than phylloquinone in PSI from axenic *Gloeobacter violaceus* cultures^13^, thereby demonstrating that these bacteria harbor a functional menaquinone biosynthesis pathway. However, these authors were unable to find the classical pathway in this species^13^. We confirm this result and have extended it to *G. lithophora* that also lacks the Men genes upstream of DHNA. We then searched in these genomes for components of the more recently discovered, alternative futalosine pathway^6^ and did not find homologs in Gloeobacterales (defined here as *Gloeobacter* + *Gloeomargarita*). These results suggest either that Gloeobacterales synthesize menaquinone through a novel pathway, or alternatively, they contain genes of the Men or futalosine pathways that have diverged beyond recognition using comparative sequence analysis. In either case, it is possible that glaucophytes and *G. sulphuraria* have gained these genes through EGT and we were unable to identify them.

### Establishment of the plant Men pathway peroxisomal bypass

In green and red algae as well as in plants, O-succinylbenzoate (OSB), the reaction product of the plastidial phyllo fusion protein is targeted to the peroxisome where it is converted, via three enzymatic steps, to DHNA (Fig. 1)^4^. Eukaryotic peroxisomes harbor benzoic acid metabolism as well as numerous CoA transferases and CoA thioesterases that could have played a role in the establishment of this segment of the pathway (reviewed in 4). The DHNA-thioesterase of green and red algae are unlikely to derive from bacterial Men pathway enzymes and could comprise undefined, low specificity CoA thioesterases involved in distinct peroxisomal pathways. We confirm, nevertheless, the previous observation^14^ that a true DHNA-CoA thioesterase from a bacterial (non-chlamydial) source was transferred later into land plants.

Because of pre-existing peroxisomal CoA transferases and thioesterases, implementation of the peroxisomal segment of the pathway would have required LGT of the only step that could not occur via eukaryotic enzymes. No peroxisomal activity could substitute for dihydroxynaphthoic acid synthetase, that is responsible for converting the single ring activated benzoate derivative, o-succinylbenzoate-CoA, into the double ring 1,4-dihydroxy-2-naphthoyl-CoA. This step was achieved through LGT and expression of the *MenB* gene (Supplementary Fig. S5). All Archaeplastida, excluding Cyanidiophytina, are monophyletic in the MenB phylogeny with strong support (BP = 1, BV = 94%). This result dates the LGT of *MenB* to Archaeplastida prior to the diversification of glaucophytes and red and green algae. The presence of MenB in the host prior to endosymbiosis is unlikely because all currently available heterotrophic eukaryotic genome sequences not derived from Archaeplastida lack Men pathway genes (with the exception of UBIAD1). As with most other Men genes, the MenB phylogeny does not allow us to identify the prokaryotic source(s) of this gene. The LGT of *MenB* at an early stage post-endosymbiosis implies that a source of o-succinylbenzoate-CoA was present in the peroxisome. This in turn implies the production of OSB in the host. Because heterotrophic eukaryotes are unable to produce this compound and do not contain Men genes other than *MenA*, OSB must either have been provided by the cyanobiont or by prior LGTs of bacterial Men genes to the nucleus of the host. Activation of OSB to o-succinylbenzoate-CoA was probably initially catalyzed by low specificity peroxisomal benzoic acid CoA transferases. Optimization of this reaction was rapidly achieved by LGT of *MenE* to the host nucleus (Supplementary Fig. S7). This also happened before diversification of the red and green algae. All *MenB* and *MenE* sequences, with the exception of the Cyanidiophytina enzymes, contain peroxisomal targeting sequences, suggesting a post-endosymbiosis, but early origin of the peroxisomal branch of plant menaquinone/phylloquinone metabolism.

**Figure 6:**
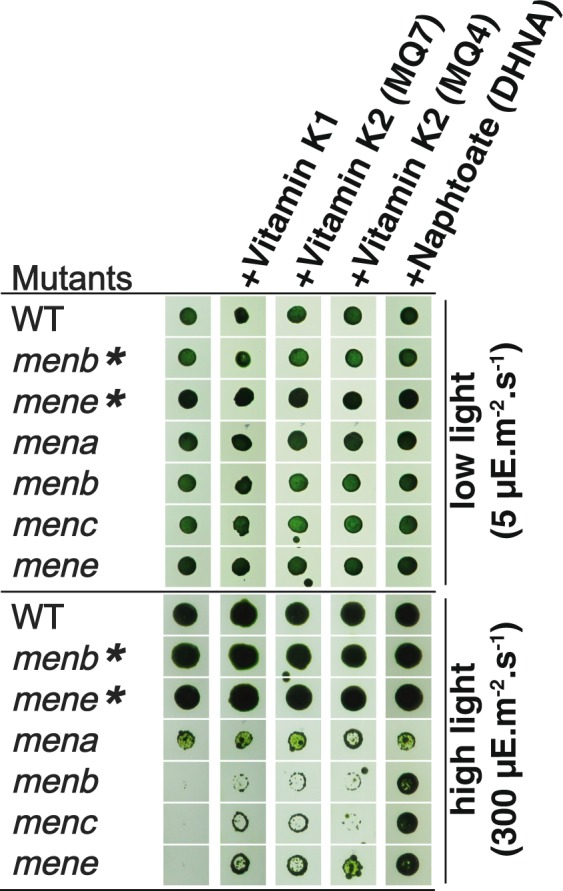
Growth of control and menaquinone mutant strains under different culture supplementation conditions. 20 μl of cell culture at 106 cells.ml-1 were spotted on classical TAP media, or supplemented media with either Vitamin K1, Vitamin K2 (MQ7), Vitamin K2 (MQ4), or Naphtoate (DHNA) and then submitted to either high or low light conditions for 7 days. The ‘*’indicate mutants rescued by supplementation.

The hypothesis of an early supply of OSB to the peroxisome from the cyanobiont requires that all of the intermediates can diffuse freely across cyanobacterial, inclusion, and peroxisomal membranes. We tested this prediction and confirm that vitamin K_1_^15^ as well as DHNA (present work) can cross both plasma and plastid membranes because they rescue light sensitivity due to Men gene mutations in *Chlamydomonas reinhardtii* (Fig. 6). In addition, mutants of *E. coli* and *B. subtilis* defective for OSB synthesis can be readily supplemented by adding OSB to the external medium^16^.

### Investigating the evolutionary history of the isoprenoid MEP pathway

The next phase of our study targeted the phylogenetic origin of genes involved in isoprenoid metabolism that is required for menaquinone/phylloquinone synthesis (Fig. 1). The non-mevalonate, bacterial MEP pathway (2-C-methyl-D-erythritol 4-phosphate/1-deoxy-D-xylulose 5-phosphate) operating in extant plastids consists of eight enzymes (Fig. 1; Supplementary Figs S15–22), including IPP isomerase (Idi) (Supplementary Fig. S22). These enzymes are required to synthesize DMAPP and IPP, that in turn are used by geranyl geranyl diphosphate synthase (GGPPS) to generate GGPP, a C_20_ terpenoid precursor for menaquinone synthesis (Supplementary Fig. S23). This molecule is further reduced by geranyl geranyl diphosphate reductase (GGPPR) for the synthesis of the phytyl chains used to generate phylloquinone (Supplementary Fig. S24). As with the Men pathway, the MEP pathway (Fig. 1) is of prokaryotic origin and absent from heterotrophic eukaryotes unrelated to Archaeplastida. Therefore, when LGTs are evident in eukaryotes but direction of transfer is unclear, we will assume a prokaryote donor. Among the 9–10 steps required to produce the prenyl substrates of the MenA transferase, two are of chlamydial origin in all three Archaeplastida lineages (e.g., CDP ME synthase (*IspD*) (Supplementary Fig. S17 with BP = 1) and CDP ME kinase (*IspE*) (Supplementary Fig. S18 with BP = 0.99), in agreement with previous findings^17–20^. A third LGT (HMBPP reductase, *IspG*) is of chlamydial origin in the green lineage (BP = 1) and of cyanobacterial origin (BP = 1) in red and glaucophyte algae (Supplementary Fig. S20). The phylogeny of IspF (encoding MEcPP synthase) excludes cyanobacteria as donors of the archaeplastidal homolog (Supplementary Fig. S19), however, the tree supports a PVC (i.e., a single sequence from the planctomycete *Phycisphaera mikurensis*) donor for the archaeplastidal homolog (BP = 0.98). In Chlamydiales the product of IspF (2-C-methyl-D-erythritol 2,4-cyclodiphosphate) is an important regulator of gene expression because it relaxes DNA compaction due to the interaction of chlamydial nucleoids with histone-like proteins^21^. This additional function appears to have led to elevated sequence divergence rates in many pathogens which is evidenced by long branches in phylogenetic trees. Among these Chlamydiales sequences, one of two *Criblamydia sequanensis* MEcPP synthase isoforms groups closely with *P. mikurensis*. However, inclusion of these long branch clades lowers the BP values in the IspF tree. It is thus possible, although a speculative idea at this juncture, that Archaeplastida gained the MEcPP gene from an ancestral Chlamydiales-like intracellular pathogen (Supplementary Fig. S25).

In addition to the enzymes of phytyl synthesis GGPPS and GGPPR, only two steps in the MEP pathway in Archaeplastida are derived from cyanobacteria DOXP reductase, (IspC; Supplementary Fig. S16) and HMBPP reductase, (IspH; Supplementary Fig. S21). Because the putative major flux-controlling step of the pathway (DXP^22^; (Supplementary Fig. S17) is not of cyanobacterial origin, our results suggest that the cyanobacteria lost control of isoprenoid synthesis soon after plastid endosymbiosis. In summary, one-third of the MEP isoprenoid synthesis pathway is of chlamydial origin in Archaeplastida. That this pathway was subject to the same types of biotic interaction as those proposed to explain the evolution of tryptophan^23^ or DHNA syntheses (this work) is supported by the loss of the cyanobacterial DOXP synthase (*Dxs*) that initially gated the flux in this pathway and was replaced through LGT by an alpha-proteobacterial enzyme (Supplementary Fig. S15).

## Discussion

Single gene phylogenies that address ancient events are particularly prone to signal erosion. Despite this concern, some of the MEP trees provide clear outcomes in terms of prokaryotic donors to Archaeplastida. Three out of 7 enzymes are of chlamydial origin (IspD, IspE, IspG). A fourth enzyme (IspF) may be of PVC origin but the rapid evolution of these sequences in Chlamydiales weakens this argument. In contrast, the greater phylogenetic uncertainty associated with Men pathway enzymes is explained by several factors. First, Men proteins are small (typically less than 350 aa) and therefore contain relatively few informative sites. A noticeable exception is MenD which is the only gene for which we recovered a robust affiliation for the Cyadiniophytina enzymes. Second, gene sharing is common among diverse bacterial clades phyla making it difficult or even impossible to predict ancient origins. The absence of congruence of phylogenetic trees (with the notable exception of MenFD) resulting from gene sharing disallows gene concatenation thereby preventing an increase of phylogenetic signal. Third, differential evolutionary forces act on plastid versus nuclear encoded loci (e.g., AT-enrichment of plastid genes), that leads to divergent phylogenetic signals and biased codon usage (e.g.^24^). Despite these limitations, we provide evidence for the chlamydial origin of the MenFD proteins in the Cyanidiophytina plastid Men cluster and argue that all 7 Men genes “arrived” together in the ancestor of this extremophilic lineage of red algae.

Absence of the classical Men pathway in extant cyanobacteria that are thought to be the closest relatives of the primary plastid suggests that the Men gene cluster may potentially have entered post-endosymbiosis. This hypothesis will be validated when the pathway of menaquinone synthesis is elucidated in these cyanobacteria. It will be exciting to investigate if, as we suspect, the genes of this novel Gloeobacterales pathway of menaquinone synthesis have been transmitted to the glaucophyte lineage. Because the maintenance of two distinct pathways of menaquinone synthesis in the free living cyanobacterial ancestor is highly unlikely, such a result would considerably strengthen evidence for the recently proposed source of the primary plastid. This result would also narrow down the timing of Men gene LGTs to a small window post-endosymbiosis but before Archaeplastida diversification at the earliest stage of the process of organelle integration.

The “ménage à trois” hypothesis (MATH)^25^ suggests a direct role for Chlamydiales obligate intracellular pathogens in plastid establishment and is supported by a wide range of phylogenetic, molecular, and biochemical lines of evidence^26^. A total of 30–100 genes have been transferred to Archaeplastida by these pathogens. This represents 3–10% of the well-accepted cyanobacterial contribution via EGT to the genomes of these photosynthetic taxa^17–20,25^. Nevertheless, it has been argued that the impact of Chlamydiales on Archaeplastida evolution is not significantly different than that of any other bacterial group, save the cyanobacteria^27^. Others have questioned the timing and relevance of these LGTs^28,29^ or interpretation of single gene phylogenies^30^. Recent data have however corroborated the role of Chlamydiales in plastid establishment^23^ and addressed many existing concerns^23,26,31–33^. We also report here a disproportionate contribution of Chlamydiales to Archaeplastida plastid functions and demonstrate that this association^31^ is strengthened with the availability of novel genome data. Using the *Arabidopsis thaliana* plastid proteome as query. This plastid proteome includes proteins encoded by both the plastome and the nuclear genome. We find that 17 of these proteins are of unambiguous chlamydial origin, compared to 6 from all proteobacterial clades, that are the third-most frequent sources of LGT to this compartment. This result holds, despite the fact that the protein database for Proteobacteria is >34-fold larger than for Chlamydiales (Supplementary Fig. S26 and Supplementary Table 1).

We demonstrate here an additional significant contribution of Chlamydiales to menaquinone metabolism in Archaeplastida. These genes impact the synthesis of both the core quinone and the isoprenoid (prenyl) side chain. Most importantly, we provide evidence that plastid genes encoding enzymes of the Men pathway in Cyanidiophytina are most likely of chlamydial (and not cyanobacterial [endosymbiont]) origin. It is presently unclear that this gene cluster was derived from an event that is either independent or shared with the bacterial LGT of the nuclear Men genes in the Archaeplastida ancestor. Future work directed towards elucidating the nature of the menaquinone synthesis pathway in Gloeobacterales and Glaucophyta will enable us to discriminate between several competing hypotheses to explain the role of Chlamydiales and other bacteria in the origin of vitamin K biosynthesis. In the discussion below, we present two alternative explanations for our results, first in light of the classical endosymbiosis theory and second, in light of the MATH.

Gene transfer to the nucleus and subsequent loss of the original endosymbiont genes does not necessarily translate into EGTs. Often, genes that were not encoded on the endosymbiont genome replace the corresponding EGT-derived gene resulting in LGT from diverse, mostly bacterial, sources^31^. These are considered as “chance” replacements with little or no functional relevance, other than possibly providing a protein substrate better suited to the complex import machinery of the evolving plastid or because the EGT process has dramatically slowed down. This may have occurred in the MEP pathway. What is unusual here is the frequency of the events. It seems that Chlamydiales-like ancestors have donated 4/8 MEP pathway genes, other bacteria having donated a single gene, whereas cyanobacteria donated 2 genes via EGT. The host supplied the final isomerase function. The high frequency of chlamydial LGTs might in turn be due to the transfer of a single region of DNA encompassing *IspD*, *IspE*, and *IspF*. Later LGT of *IspG* occurred in the green lineage, whereas red algae and glaucophytes had the expected EGT of the corresponding cyanobacterial genes. This view of LGT or EGT argues against a specific role for Chlamydiales in the initial events of plastid endosymbiosis. The presence of 3–4 genes of possible chlamydial origin can be viewed here as entirely circumstantial. As to the menaquinone core synthesis genes, the possible absence of the Men pathway genes in basal cyanobacteria would suffice to explain the prevalence of bacterial LGTs. A limited number of LGTs, possibly of gene clusters from a single bacterial source, thereby generating protein fusions, would have allowed synthesis of OSB in the plastid that could have been directly used by the peroxisomes if an additional single successful LGT (*MenB*) had occurred. The origin of the plastid Men cluster in Cyadiniophytina, under the classical endosymbiotic theory can be viewed as an additional exceptional and entirely circumstancial event, whereby a chlamydial gene cluster was transferred to the plastid genome of these single-celled red algae. This was accompanied by higher levels of expression of menaquinone metabolism enzymes in the plastid and led to the loss of the nuclear phyllo Men protein fusion. This may have happened as a result of natural selection when the currently hot spring-dwelling Cyanidiophytina adapted to warmer environments with lower oxygen availability.

According to the MATH, Chlamydiales had developed, prior to plastid endosymbiosis, a set of complex biotic interactions whereby they sequestered a cyanobacterium in their inclusion and enslaved it to extract photosynthetic carbon as well as other components needed for replication. In order to achieve this, they genetically engineered the cyanobiont with genes donated through conjugation. These genes included transporters not encoded by cyanobacteria, genes that allowed cyanobacterial survival in the intracellular environment, and genes that increased the synthesis of compounds useful to the pathogens, such as amino acid and vitamins. Maintenance of the tripartite interaction (Chlamydia/cyanobacterium/host) was achieved by secretion of cytosolic protein effectors encoded by Chlamydiae. The onset of plastid endosymbiosis under the MATH can be viewed as the escape of the cyanobacterium from the chlamydial inclusion. Because of the prior chlamydial interaction, such a cyanobiont would have been pre-adapted to the intracellular environment, potentially explaining the rarity of primary plastid endosymbiosis. Under this view, the chlamydial phylogenomic imprint in the archaeplastidal genome consists of two very different components. The first is no different from the one discussed above under the classical endosymbiosis theory. “Chance” gene replacements may have occurred, and their frequency may have increased if the pathogens stayed “persistent” in their host for an extended period of time. The chlamydial contribution to this component need not be monophyletic and several chlamydial clades, in addition to the ones involved in the tripartite interaction could have generated this signal. In addition, the timing of “chance” replacements may be such that most of these replacements could occur in a clade-specific fashion, rather than leading to LGTs shared by the three Archaeplastida lineages. We do not expect these replacements to specifically target any particular pathway, nor are they expected to be particularly more abundant than LGTs from other bacteria in the pathways they impact. However, we expect a second component in chlamydial LGTs. These would consist of those genes that were transferred by the pathogens to the cyanobiont during the chlamydia-cyanobacteria initial biotic interaction. The signal generated would have been enhanced by EGT (rather than LGT) from the chimeric plastid genome to the nucleus of the host. This signal component would in such cases be monophyletic and restricted to the chlamydial clade responsible for the initial interaction.

These types of chlamydial contributions to plastid pathways comprise a burst of LGTs that differs by a number of features from the standard “drip-drip” model of chance replacements of cyanobacterial genes during the process of plastid genome reduction. First, several biochemical steps are impacted in all three well-studied cases, including tryptophan metabolism^23^, isoprenoid metabolism, and menaquinone core synthesis (this work) by LGTs from an environmental chlamydial cell. This suggests either transfer of complete gene clusters or operons to the cyanobiont plastid genome (menaquinone core or tryptophan metabolism) or extensive transfers of scattered chromosomal genes during bacterial conjugation (isoprenoid metabolism). Second, in all cases, the major flux-controlling steps provide evidence for the early loss of metabolic control by the cyanobiont because they include LGTs from Chlamydiales (*MenF*), from PVCs (anthranilate synthase alpha subunit *TrpE* and *IspF*), or other bacteria (DOXP synthase). Finally, whereas many pathway steps are LGT contributions to the ancestor of the three Archaeplastida lineages, some may still appear to be lineage specific transfers with the remaining genes being of cyanobacterial EGT origin. We suggest that direct transfer of genes to the cyanobiont genome in the chlamydial inclusion would have been followed by the persistence of both chlamydial and cyanobacterial gene copies in a merozygote because the cyanobacterial proteins were involved in other essential interactions. Maintenance of merozygote genes would explain differential segregation of genes in Archaeplastida lineages post-divergence, due to local selective forces acting on these taxa. Our present work establishes that Chlamydiae have donated gene clusters to early diverging eukaryotic algae at least as far back as Cyanidophytina diversification. What remains to be demonstrated more clearly is the relationship (if any) between the Cyanidophytina cluster and the phyllo protein fusions of mesophilic red and green algae.

Enough is presently known about chlamydial biology to explain the molecular function of some aspects of the hypothesized chlamydia-cyanobacterium biotic interaction. For instance, tryptophan, the costliest to synthesize of all amino acids, is one of the most important cues dictating the replication mode of Chlamydiae^34^. Animals have been demonstrated to react to chlamydial infection by inducing at least two types of tryptophan starvation responses in cells infected by these pathogens^35,36^. In addition, the proteomes of Chlamydiaceae and Chlamydiales have been demonstrated to be under selection for high or low TRP content, depending on the genes required for active replication or persistence, respectively^37^. In such a context, we have proposed that the abundance of chlamydial LGTs to Archaeplastida in tryptophan metabolism followed the direct transfer of the Chlamydiales TRP operon to the enslaved cyanobacterium^23,26^. These transfers were selected under the chlamydia-cyanobacterium biotic interaction to maximize the output of tryptophan synthesis in the cyanobacterium for pathogen benefit. However, for this increase in tryptophan synthesis to be beneficial, the amino acid must be exported to the chlamydial inclusion lumen. The finding of TyrP, the major tryptophan transporter of Chlamydiae as a chlamydial LGT common to all three Archaeplastida clades considerably strengthens this speculation^23,26^. In the case of menaquinone synthesis, no transporter seems to be required and vitamin K and intermediates of menaquinone synthesis such as OSB or DHNA readily cross bacterial or plastid membranes. Synthesis of menaquinone from chorismate is also an energetically costly pathway. Hence a boost in vitamin K synthesis in the enslaved cyanobacterium would have been immediately beneficial to the pathogen. For this to be effective, both the synthesis of the core quinone and its isoprenoid side chain must have been coordinated. We thus infer that the chlamydial Men and MEP genes were induced through direct gene transfer of chlamydial genes to the cyanobacterial genome. It is clear to us that the Chlamydiae grow better under microaerophilic conditions^38,39^ and that in this context, the pathogens only make use of menaquinone as the sole quinone used for respiration.

A recent study reported the selective expression by green algal endosymbionts of a hypoxia stress response, as they penetrate the cytosol of salamander embryo cells to establish a rare case of phototroph-vertebrate cell endosymbiosis^40^. Hence, free-living cyanobacteria that have been enslaved in a chlamydial inclusion would likely experience even greater severe hypoxic stress. Free-living cyanobacteria also contain high affinity terminal oxidases to manage such stresses in the extracellular environment^41^. Under this condition, a large increase in menaquinone synthesis would have been mutually beneficial for both Chlamydiae and cyanobacteria because this would have protected the latter from ATP starvation in darkness. NTTs, the major ATP import protein of Chlamydiae and plastids are derived from a chlamydial LGT in all three Archaeplastida lineages (reviewed in Cenci *et al*. 2017^26^). ATP homeostasis is likely to have been a severe issue for cyanobacteria that were induced to export photosynthates, and therefore depleted their carbon stores. This could have formed the basis of host-pathogen biotic interactions, whereby Chlamydiae would have induced an increased flux of menaquinone synthesis in the cyanobiont by conjugation of its operon and genes.

## Methods

### Phylogenetic analysis

We searched for genes encoding menaquinone biosynthesis enzymes by using several conserved protein sequences as BLAST queries against the following groups of interest: Rhodophyceae, Chloroplastida, Glaucophyta, Chlamydiae, and Cyanobacteria. In those organisms that did not yield homologs in these searches and that presumably lacked the standard Men pathway, we further checked their absence by using ghostkoala (http://www.kegg.jp/ghostkoala/) and thereby generating a KEGG annotation to make sure that we retrieved all possible Men pathway enzymes. We then double-checked for the presence of the expected catalytic domains in sequences retrieved by BLAST using the Interpro database (https://www.ebi.ac.uk/interpro/). For proteins containing several catalytic domains (e.g., the PHYLLO fusion), we used the interpro annotation to separate the different domains and perform the phylogenetic analysis described below.

For phylogenetic analyses we used this previous set of curated sequences to perform BLAST analysis against sequences obtained from different sources (e.g., MMETSP, NCBI) without applying any filters or clade preference. We aligned all sequences selected with an *E*-value less than 1e-10 using MAFFT with high speed settings^42^. Preliminary trees were generated with Fasttree^43^ after using BMGE^44^ with a block size of 4 and the BLOSUM30 similarity matrix. We applied a ‘dereplication’ step with TreeTrimmer^45^, sizing down robustly supported monophyletic clades in order to reduce sequence redundancy. The final set of sequences were selected manually. When we considered a set of sequences to be of sufficient high quality we re-aligned with MUSCLE, carried out a block selection using BMGE with a block size of four and the matrix BLOSUM30. The trees were generated using Phylobayes^46^ under the CAT + GTR model^47^. These models take site-specific evolutionary properties (such as rate and profile) into consideration and generally fit sequence data significantly better than one-matrix models (e.g., LG). For all of the analyses, we set up two chains that run in parallel and assessed convergence using the ‘bpcomp’ and ‘tracecomp’ functions available in Phylobayes 3.3. Convergence assessments were carried out based on sampled trees and parameters (taking one from every 10 trees) following burnin equal to 20–25% of the entire length of the chain. The consensus trees were generated with the ‘bpcomp’ function. We considered a phylogenetic analysis to be acceptable when maxdiff <0.3 and effective size >50 for all parameters, according to the Phylobayes user manual. The consensus tree was then used to map bootstrap support values from 100 replicates using IQ-TREE^48^ under the CX(10 to 30) models^49^, depending on the number of sites in each alignment. For model selection we followed a rule of thumb where the X value is around ten times under the number of the aligned dataset amino acid positions in order to avoid parameterization overfitting. For concatenation analysis we selected taxa according to single gene phylogenies performed for MenD and MenF, we aligned separately sequences from both proteins with MUSCLE, selected the block with BMGE (using the same settings as previously described), and construct phylogeny with IQ-TREE. We, then, compared trees when topology for a taxon was identical in both trees we concatenated sequences in order to improve phylogenetic reconstruction. Finally, we performed the phylogeny using IQ-TREE and the same settings as previously explained for single gene phylogeny. In addition, we used Phylobayes and took stringent parameters, considering convergence when a maxdiff <0.1 was obtained based on sample trees (taking one from every 100 trees) with a minimum effective size of at least 300 trees.

### Re-assessing the chlamydial contribution to the plastid proteome in *Arabidopsis thaliana*

Protein sequences in RefSeq (version 69) were downloaded from the NCBI FTP site (ftp://ftp.ncbi.nlm.nih.gov/refseq/). When sequences were available from more than one (sub) species in a genus (e.g., *Arabidopsis thaliana* and *A. lyrata* in the genus *Arabidopsis*), the species (e.g., *A. thaliana*) with largest number of sequence were retained, whereas others (e.g., *A. lyrata*) were removed. We incorporated all available Chlamydiae protein sequences downloaded from NCBI Genbank (May, 2017) and red algal sequences from *Porphyridium purpureum*^50^ and from MMETSP database^51^ (e.g., *Rhodosorus marinus*). To reduce sequence redundancy in the database, we clustered highly similar sequences (identity ≥85%) among taxa from each phylum (e.g., Chlamydiae and Alphaproteobacteria), retained the longest sequence and removed all other related sequences in the same cluster using CD-HIT v4.5.4^52^. This step enhanced sequence diversity from a given phylum by avoiding sequence sampling from closely related taxa.

Phylogenomic analysis was carried out as described previously^53^. *A. thaliana* plastid proteomes collected in a previous study^31^ were used as query to search against the aforementioned database using BLASTp (*e*-value cut-off = 1e-05). The top 1,200 significant hits (sorted by bit score) for each query sequence were recorded. Representative sequences were selected (allowing up to 6 sequences for each phylum) on a first-come-first-serve basis to create a taxonomically diverse sample. The BLASTp hits were then re-sorted by sequence identity (of the query-hit alignment) followed by representative sequence sampling in the same way. The two sets of representative sequences, together with the query, were then aligned using MUSCLE version 3.8.31^54^ under default settings and trimmed using TrimAI version 1.2^55^ in an automated mode (-*automated1*). The resulting alignments containing at least three bacterial sequences were used to build phylogenetic trees using IQtree^48^. The best-fit evolutionary model for each alignment was selected with the build-in model selection function^56^ and was used for the analysis. Branch support was estimated using the ultrafast bootstrap (UFboot) approximation approach^57^ with 2,000 maximum iterations (-nm 2000) and 2,000 bootstrap replicates (-bb 1500).

To infer the number of Chlamydiae-derived genes, we searched for *A. thaliana* plastid query sequences that are monophyletic with chlamydial sequences (UFboot ≥90%) allowing interruption by Archaeplastida (Viridiplantae, Rhodophyta, and Glaucophyta; and other lineages that contained a plastid of secondary endosymbiotic origin). Considering the prevalence of LGT among Bacteria, this monophyletic group was allowed to be interrupted by not more than two sequences derived from other bacterial taxa. The resulting trees were manually inspected for putative cases of chlamydial-derived LGT. LGTs derived from other sources (e.g., Alphaproteobacteria) were inferred in the same way. We encountered many instances where Archaeplastida sequences formed a monophyletic group with a bacterial phylum that was represented by a single sequence (e.g., Deltaproteobacteria represented by a *Plesiocystis pacifica* sequence instead of a clade comprising two or more Deltaproteobacteria sequences). These cases were not retained given the prevalence of LGT among bacteria and the remote possibility that the given gene was lost in all other lineages of the phylum. Gene trees (due to recent gene duplication in *Arabidopsis thaliana*) associated with the same LGT case were grouped manually. The resulting non-redundant (or independent) LGT instances were counted and reported in Table S1.

Compared to our previous report^31^, there is a minor change in Chlamydial-derived proteins. The number of *A. thaliana* proteins derived from proteobacterial phyla decreased dramatically primarily because the donor phylum was represented by a single sequence in many cases. Note that the extent of sequence sampling is much higher in Proteobacteria than in Chlamydiae (Supplementary Fig. S4). The contribution of Chlamydiae to *A. thaliana* is more pronounced when considering its minimal sequence number in our database when compared to Proteobacteria (Supplementary Fig. S4).

### Metabolite supplementation of the light sensitivity phenotype of *Chlamydomonas* menaquinone synthesis deficient mutants

The wild-type strain as well as the menaquinone mutants used for growth analysis were cultivated into liquid TAP media at 25 °C under continuous light, for 4 days^16^. For each strain, we spotted 20 µl of cell culture at 10^6^ cell.ml^−1^ on solid TAP media (1.5% agar) supplemented either with 5 mM each of Vitamin K1, Vitamin K2 (MQ7), Vitamin K2 (MQ4), Naphtoate (DHNA) or o-succinyl-benzoate (OSB). We then submitted cultures to either high (300 μE.m^−2^.s^−1^) or low (5 μE.m^−2^.s^−1^) light for 7 days.

### Characterization of naphtoquinones in microalgae

Pigments were extracted from *Chlamydomonas reinhardtii* and *Cyanophora paradoxa* lyophilized cells with N,N-dimethylformamide^58^. The resulting extracts was applied to an Acquity UPLC BEH C18 column (1.7 µm, 2.1 mm × 150 mm, Waters, http://www.waters.com/) at 40 °C and were separated at a flow rate of 0.3 ml min-1 using an UPLC (Acquity UPLC I-Class system, Waters) coupled with tandem mass spectrometry (Q Exactive, Thermo Scientific). The elution liquid gradient was generated by the following steps using solvent A (formic acid: water = 0.1: 99.9) and solvent B (methanol): 0–5 min, 15% A/85% B to 5% A/95% B, hold to 9 min; 9–10 min, 5% A/95% B to 100% B, hold to 11.5 min; 11.5–12 min, 100% B to 15% A/85% B. The injection volume was 1 µl. Mass spectroscopy measurements were performed in a positive mode ESI. The elution profile of the *C. paradoxa* cell extract was compared with the elution profile of the *C. reinhardtii* cell extract. In *C. reinhardtii*, 90% of the total amount of naphthoquinones is present as OH-PhQ^59^. OH-PhQ, whose determined m/z values are 467.35 and 489.33 for the non-adduct form and Na+ adduct form, respectively, was detected as a single peak at ~7.6 min of the chromatogram (selected monitoring mode, m/z = 466.5–467.5). This peak was missing in the *C. reinhardtii* men mutant cell extracts impaired in various steps of the phylloquinone biosynthesis pathway^60^.

## Electronic supplementary material

Dataset 1
