# Supplementary material for: Host-pathogen biotic interactions shaped vitamin K metabolism in Archaeplastida
Source: Sci Rep. 2018 Oct 15;8:15243. doi: 10.1038/s41598-018-33663-w (PMC6189191; doi:10.1038/s41598-018-33663-w)
Supplement: Supplementary file 1 — Dataset 1 [file 41598_2018_33663_MOESM1_ESM.pdf]

Title: Host-pathogen biotic interactions shaped vitamin K metabolism in Archaeplastida

Authors:

Cenci U[1+]; Qiu H[2+], Pillonel T[3], Cardol P[4], Remacle C[4], Colleoni C[1], Kadouche D[1], Chabi M[1], Greub G[3], Bhattacharya D[5,+], Ball, SG[1+]*

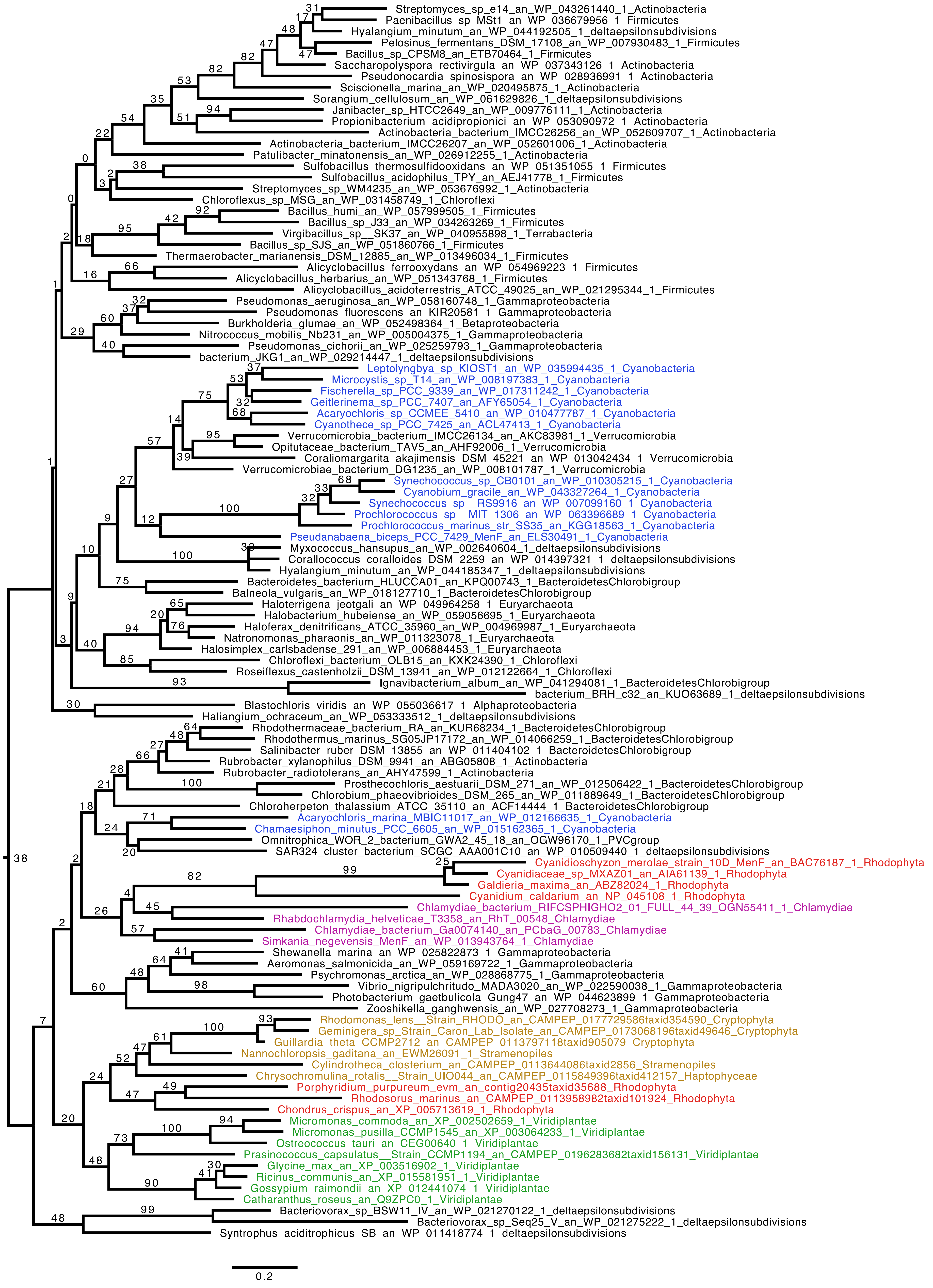

Supplementary figure S1. Phylogenetic analysis of the plastidial Cyanidiophytina MenF genes. The tree shown was obtained with IQ-TREE (Nguyen et al. 2015) obtained with the C20 model, boostrap values are displayed onto the nodes. The tree is midpoint rooted. Sequences are colored according to their taxonomic affiliation: Chlamydiae (purple), Cyanobacteria (dark blue), Viridiplantae (green), Rhodophyta (red), and algae bearing a plastid obtained by secondary or tertiary plastid endosymbiosis (brown). Other organisms are in black. The scale bar shows the inferred number of amino acid substitutions per site. MenF from the Cyanidiophytina and Chlamydiae show a common origin with a low BV of 26.

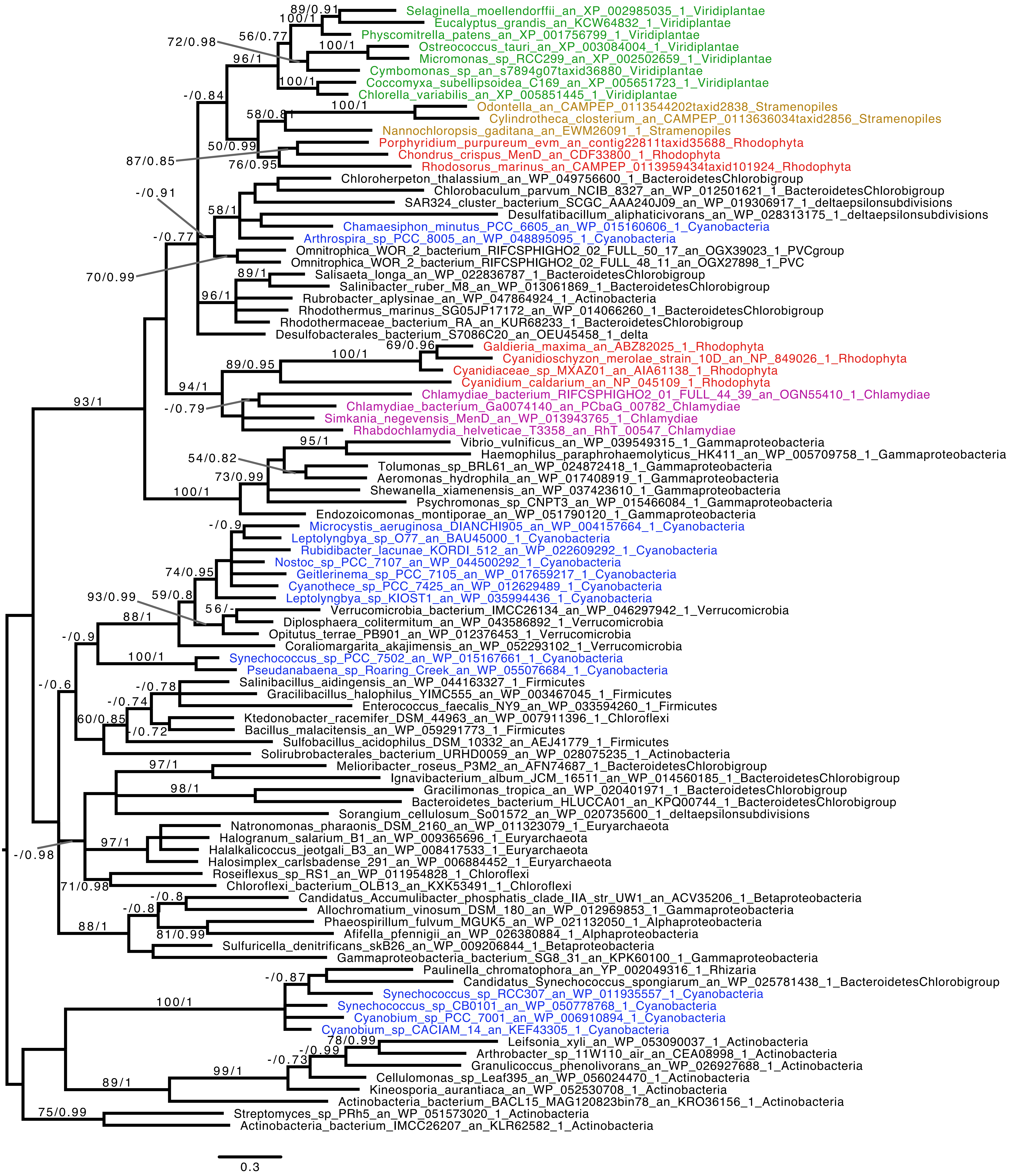

Supplementary figure S2. Phylogenetic analysis of the plastidial Cyanidiophytina MenD genes. The trees shown are the consensus trees obtained with Phylobayes (Lartillot et al., 2009) under the CAT+GTR models (Lartillot and Philippe, 2004) with ML boostrap values obtained with the C20 (left) and Bayesian posterior probabilities (right) mapped onto the nodes. Bootstrap values (BV) >50 % are shown, while only posterior probabilities (BP) >0.6 are shown. The trees are midpoint rooted. Sequences are colored according to their taxonomic affiliation: Chlamydiae (purple), Cyanobacteria (dark blue), Viridiplantae (green), Rhodophyta (red), and algae bearing a plastid obtained by secondary or tertiary plastid endosymbiosis (brown). Other organisms are in black. The scale bar shows the inferred number of amino acid substitutions per site. MenD from the Cyanidiophytina and Chlamydiae show a common origin with a BP of 1 and a BV of 94.

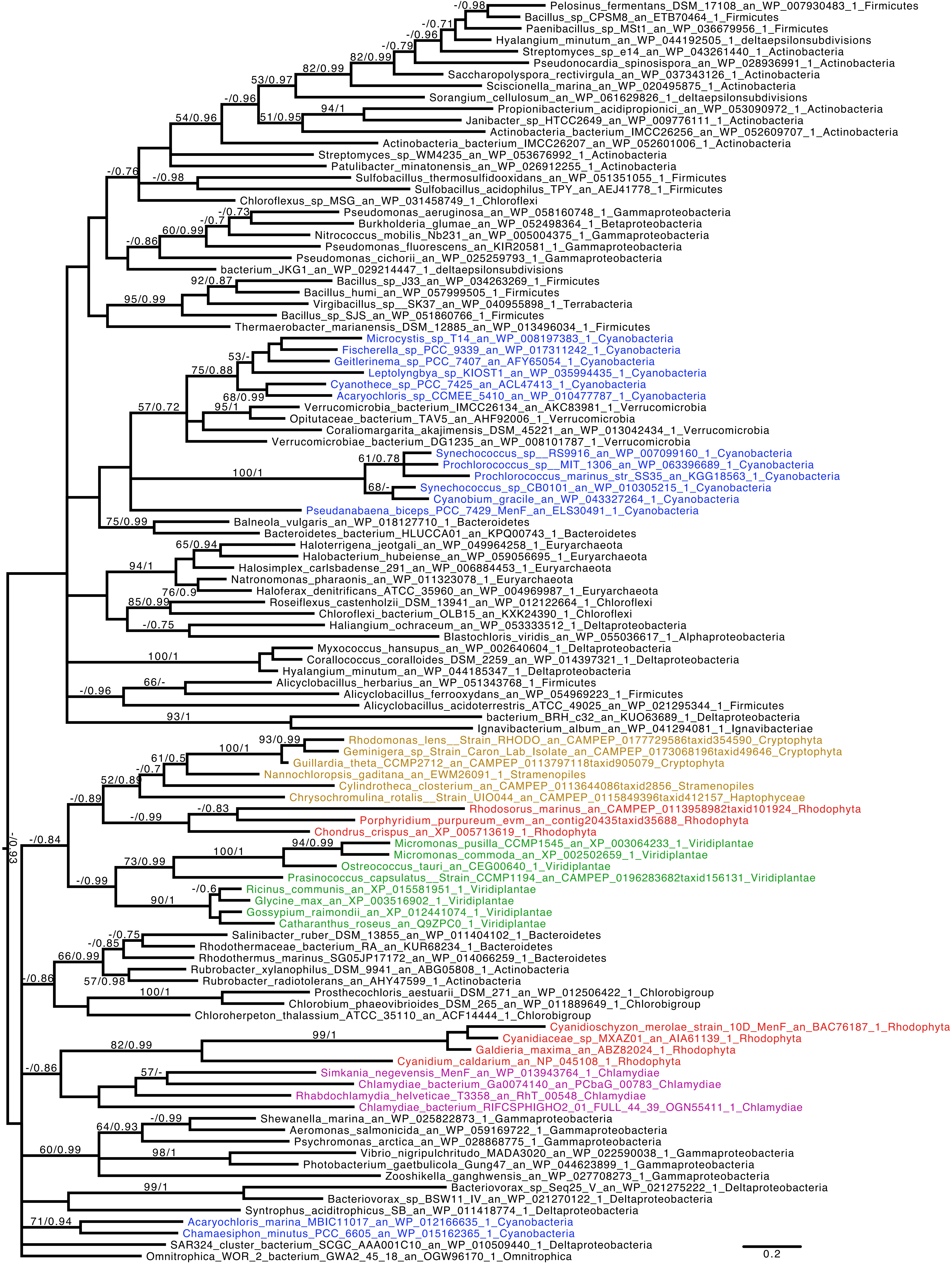

Supplementary figure S3. Phylogenetic analysis of the plastidial Cyanidiophytina MenF genes. The trees shown are the consensus trees obtained with Phylobayes (Lartillot et al., 2009) under the CAT+GTR models (Lartillot and Philippe, 2004) with ML boostrap values obtained with the C20 (left) and Bayesian posterior probabilities (right) mapped onto the nodes. Bootstrap values (BV) >50 % are shown, while only posterior probabilities (BP) >0.6 are shown. The trees are midpoint rooted. Sequences are colored according to their taxonomic affiliation: Chlamydiae (purple), Cyanobacteria (dark blue), Viridiplantae (green), Rhodophyta (red), and algae bearing a plastid obtained by secondary or tertiary plastid endosymbiosis (brown). Other organisms are in black. The scale bar shows the inferred number of amino acid substitutions per site. MenF from the Cyanidiophytina and Chlamydiae show a common origin with a BP of 0.86 and a BV of 26 (not indicated).

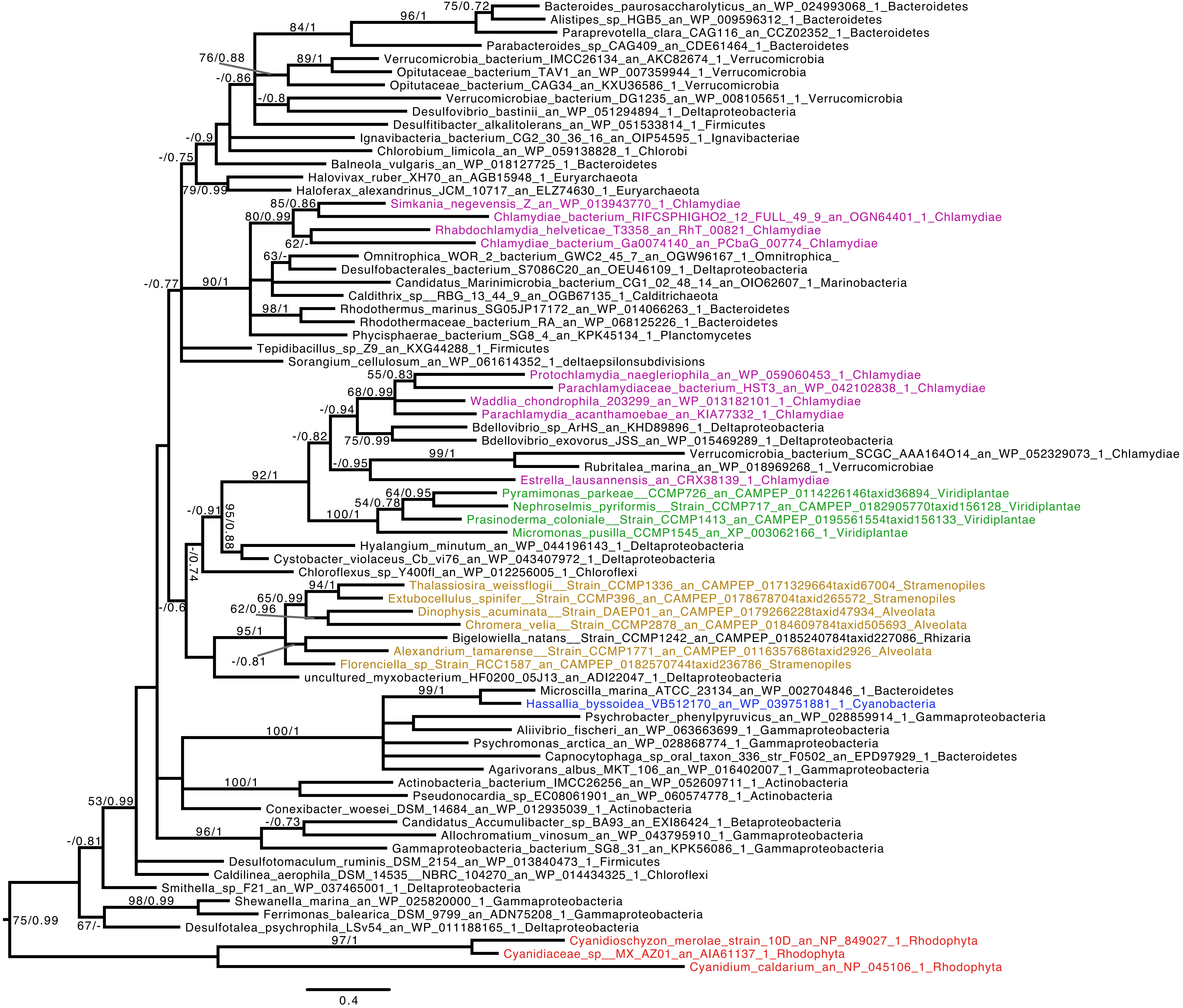

Supplementary figure S4. MenA with emphasis on the Cyanidiophytina and Chlamydiae (Archaeplastida are displayed in supplementary Fig 12) phylogenetic analysis from the plastidial Cyanidiophytina Men cluster genes. The trees shown are the consensus trees obtained with Phylobayes (Lartillot et al., 2009) under the CAT+GTR models (Lartillot and Philippe, 2004) with only ML bootstrap values (BV) >50 % obtained with C20 model (left) shown, and Bayesian posterior probabilities with only those (BP) >0.6 shown (right). The trees are midpoint rooted. Sequences are colored according to their taxonomic affiliation: Chlamydiales (purple), Cyanobacteria (dark blue), Rhodophyta (red), algae bearing a plastid obtained by secondary or tertiary endosymbiosis (brown), and Glaucophyta (light blue). Other organisms are in black. The scale bar shows the inferred number of amino acid substitutions per site. This phylogenetic tree rejects cyanobacteria but not Chlamydiae as donors. They however remain unresolved.

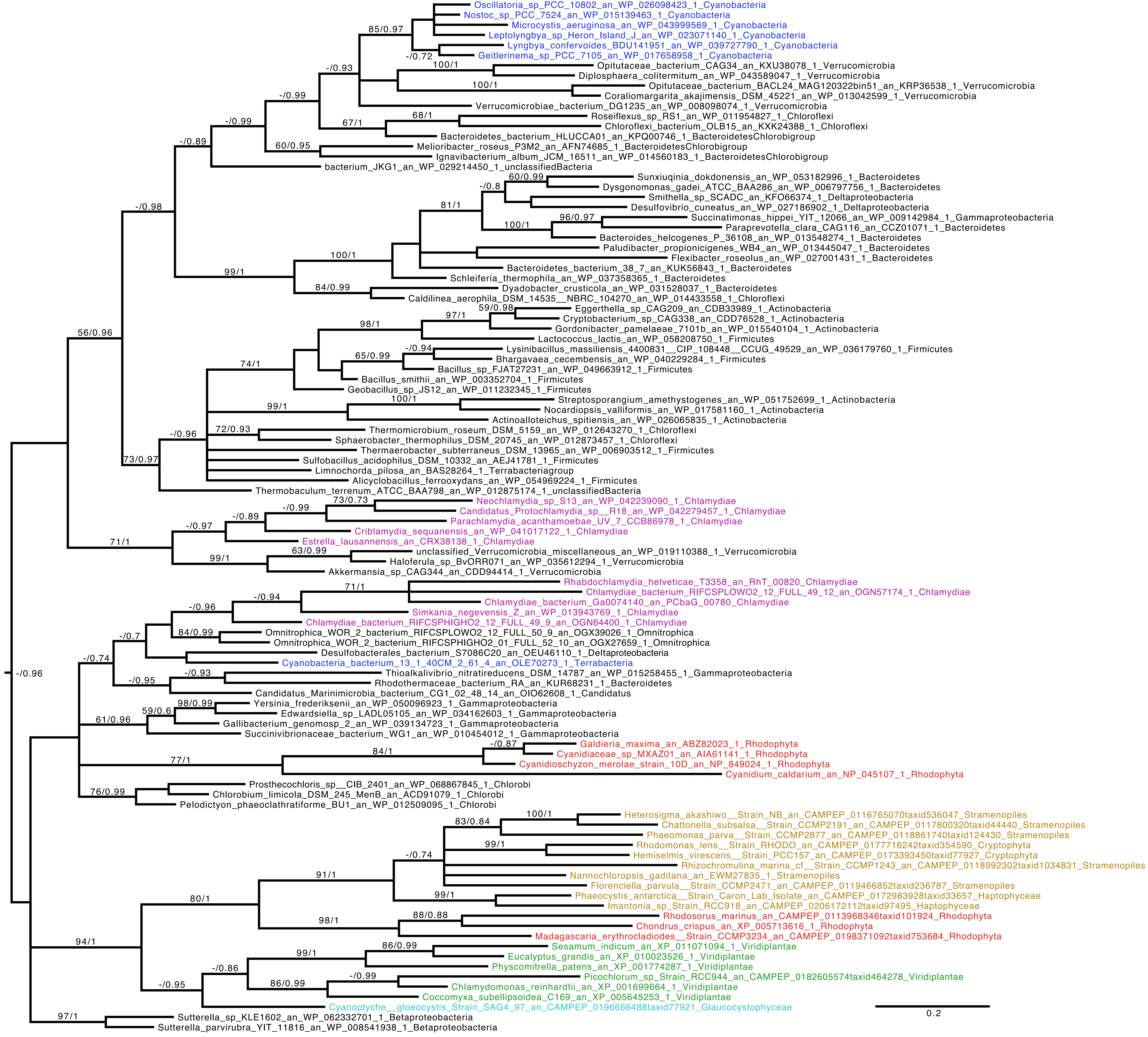

Supplementary figure S5. MenB with emphasis on Cyanodiphitina, Archaeplastiida and Chlamydiae phylogenetic analysis from the plastidial Cyanidiophytina Men cluster genes. The tree was obtained and is displayed as in supplementary figure 4. This phylogenetic tree rejects cyanobacteria but not Chlamydiae as donors. They however remain unresolved.

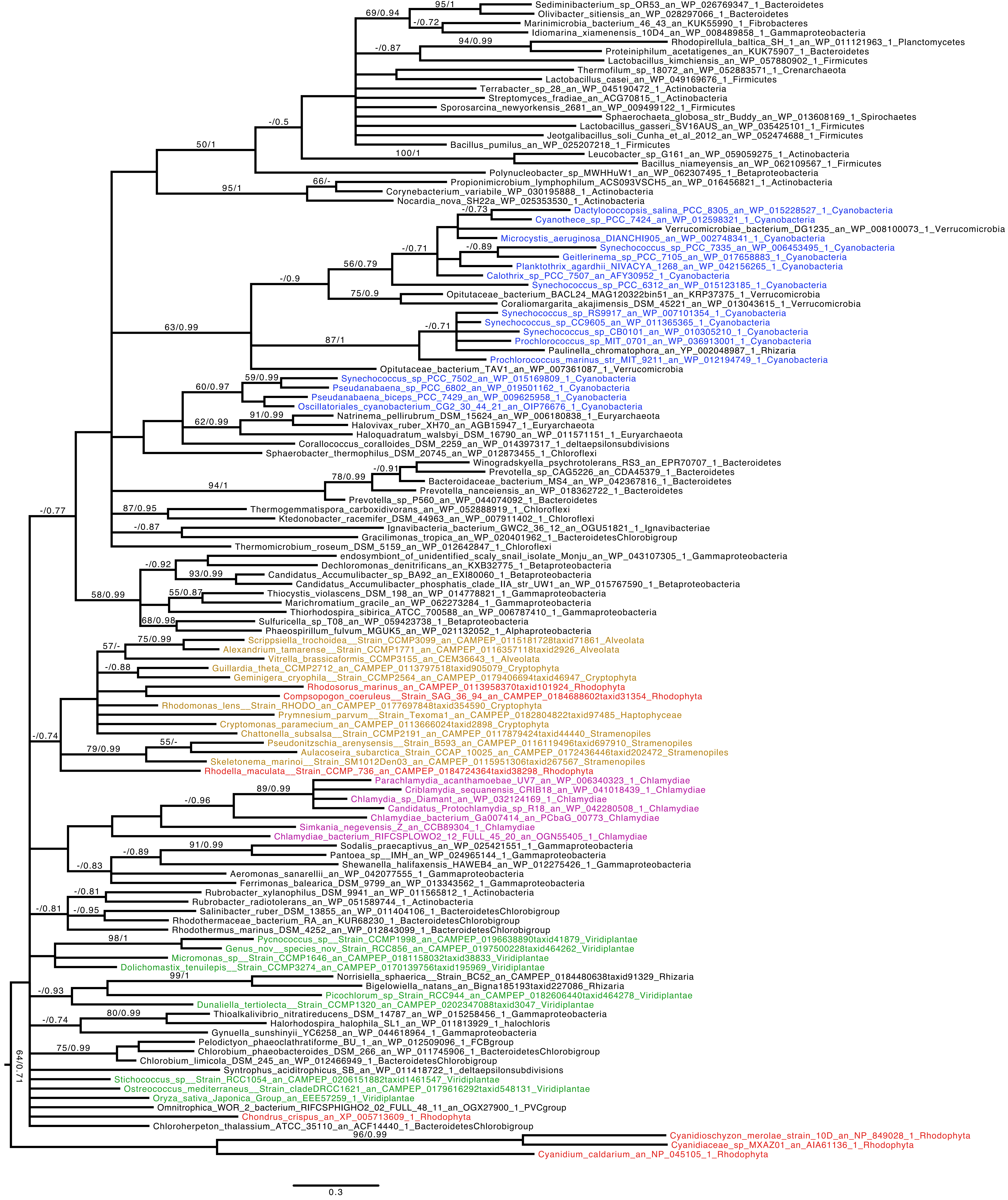

Supplementary figure S6. MenC with emphasis on Cyanidiophytina, Archaeplastida and Chlamydiae phylogenetic analysis from the plastidial Cyanidiophytina Men cluster genes. The tree was obtained and is displayed as in supplementary figure 4. This phylogenetic tree rejects cyanobacteria but not Chlamydiae as donors. They however remain unresolved.

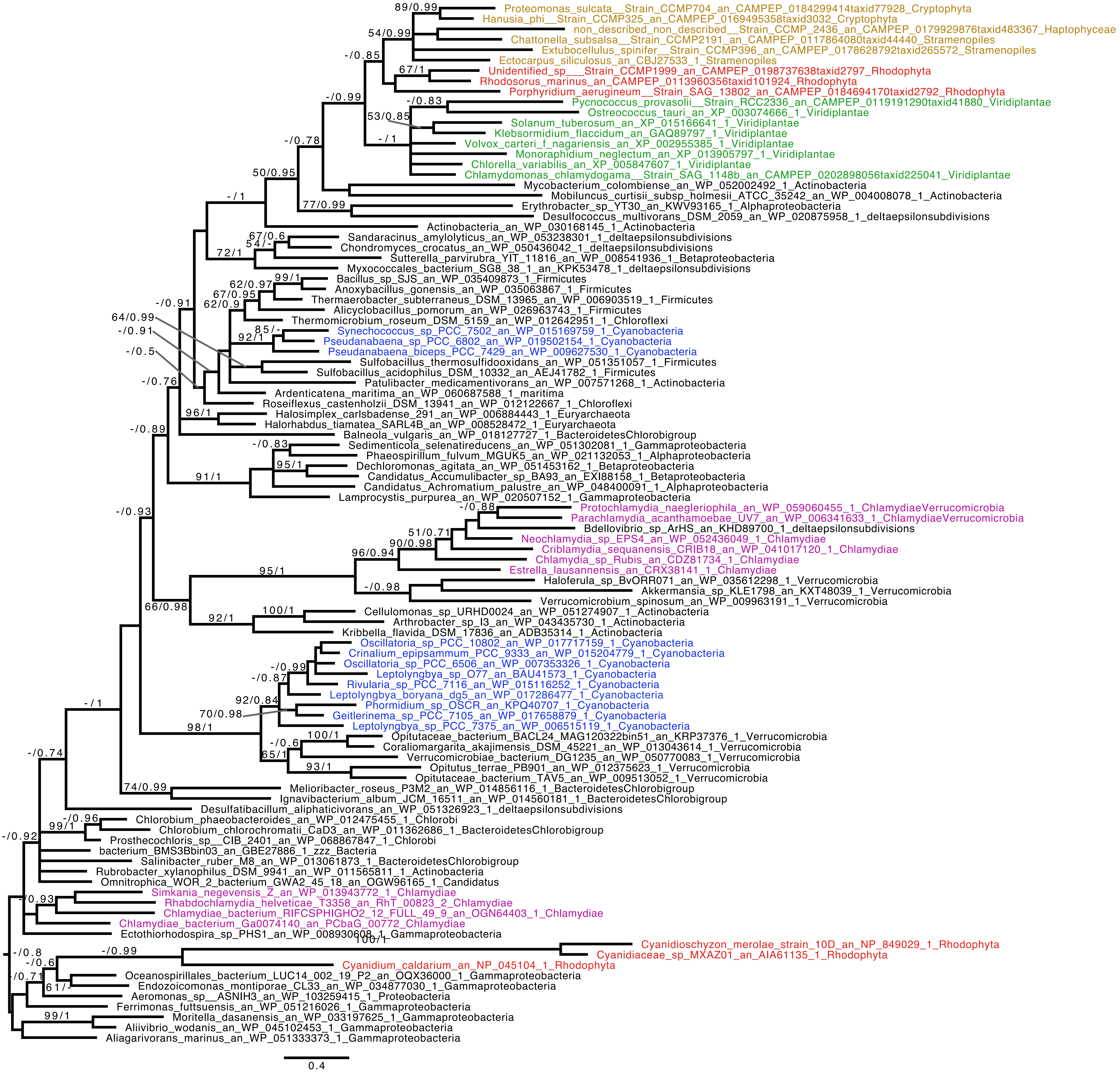

Supplementary figure S7. Phylogenetic analysis from the plastidial Cyanidiophytina MenE genes. The tree was obtained and is displayed as in supplementary figure 4. Topopology of the MenE tree is unclear, but does not reject a chlamydial origin in both Baysian and ML tree topologies.

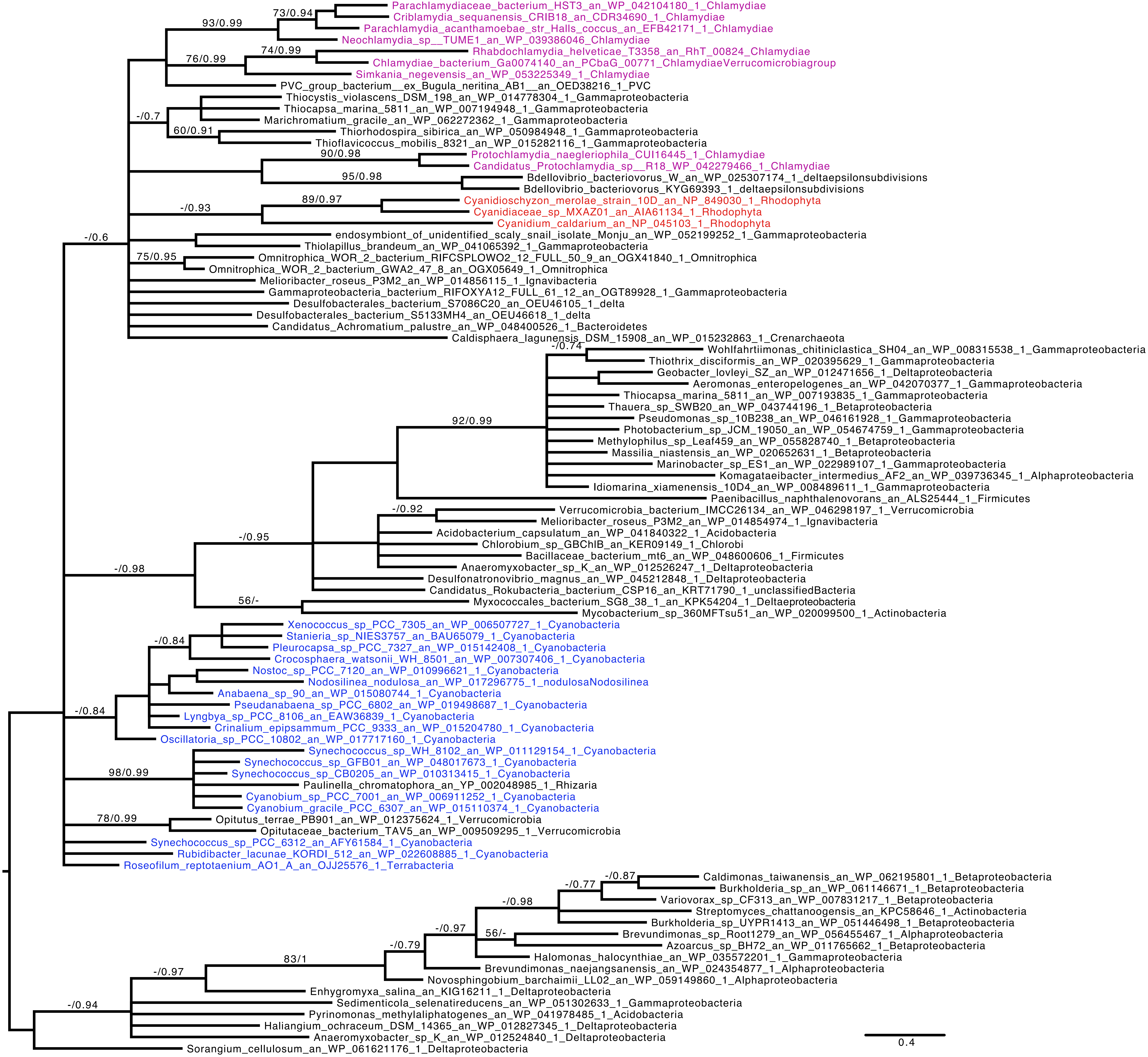

Supplementary figure S8. DHNAT with emphasis on Chlamydiae and Cyanidiophytina (land plants are affiliated to bacteria in another section of the tree while red and green algae do not contain bacterial-like DHNAT sequences) phylogenetic analysis from the plastidial Cyanidiophytina Men cluster genes. The tree shown is the consensus tree obtained with Phylobayes (Lartillot et al., 2009) under the CAT+GTR models (Lartillot and Philippe, 2004) with only ML bootstrap values (BV) >50 % obtained with C10 model (left) shown, and Bayesian posterior probabilities with only those (BP) >0.6 shown (right). The trees are midpoint rooted. Sequences are colored according to their taxonomic affiliation: Chlamydiales (purple), Cyanobacteria (dark blue), Rhodophyta (red), algae bearing a plastid obtained by secondary or tertiary endosymbiosis (brown), and Glaucophyta (light blue). Other organisms are in black. The scale bar shows the inferred number of amino acid substitutions per site. This phylogenetic tree rejects cyanobacteria but not Chlamydiae as donors. They however remain unresolved.

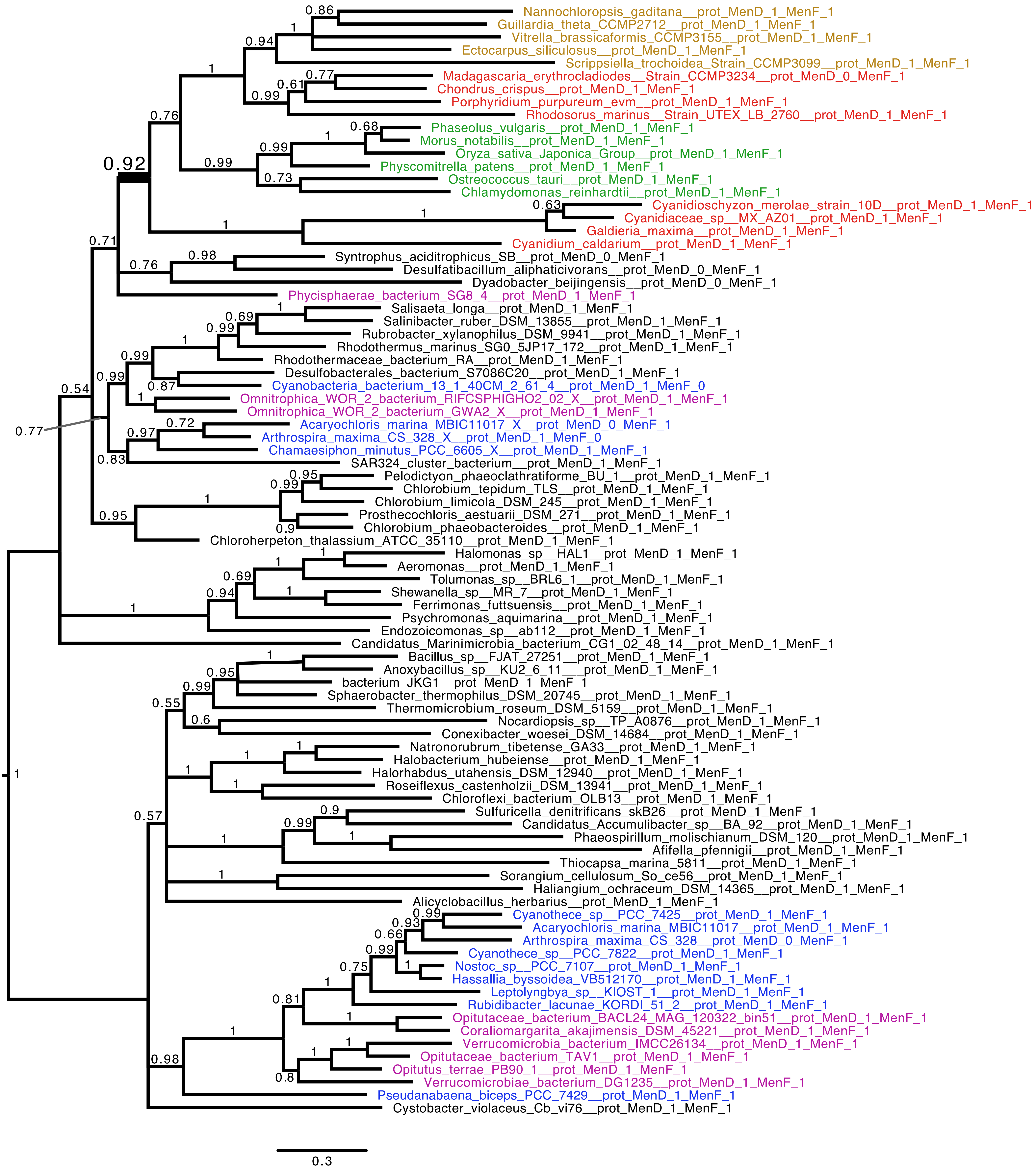

Supplementary figure S9. Concatenated phylogenetic analysis of MenFD without Chlamydiales. The tree displayed was obtained by deleting the sequences from the same alignment than the one used in Figure 3 and 4, and built using phylobayes (Lartillot et al., 2009) under the CAT+GTR models (Lartillot and Philippe, 2004). We used stringent criteria and we considered convergence when maxdiff < 0.1 were obtained based on sample trees (taking one from every 100 trees) after a minimum of at least 300 trees. We assess the burn out to be of 130. Trees are rooted on midpoint. The scale bar shows the inferred number of amino acid substitutions per site. The Rhodophyta are displayed in red, Chloroplastida are stained in green, algae bearing a plastid obtained by secondary or tertiary plastid endosymbiosis are shown in brown, all other taxa are displayed in black. If Chlamydiales are removed, we could retrieve the monophyly of the Archaeplastida with the CAT+GTR model and significantly increase the bootstrap support for Archaeplastida monophyly, even if the statistical support of the nodes fails short of the required values to strongly support this conclusion (BP 0.92).

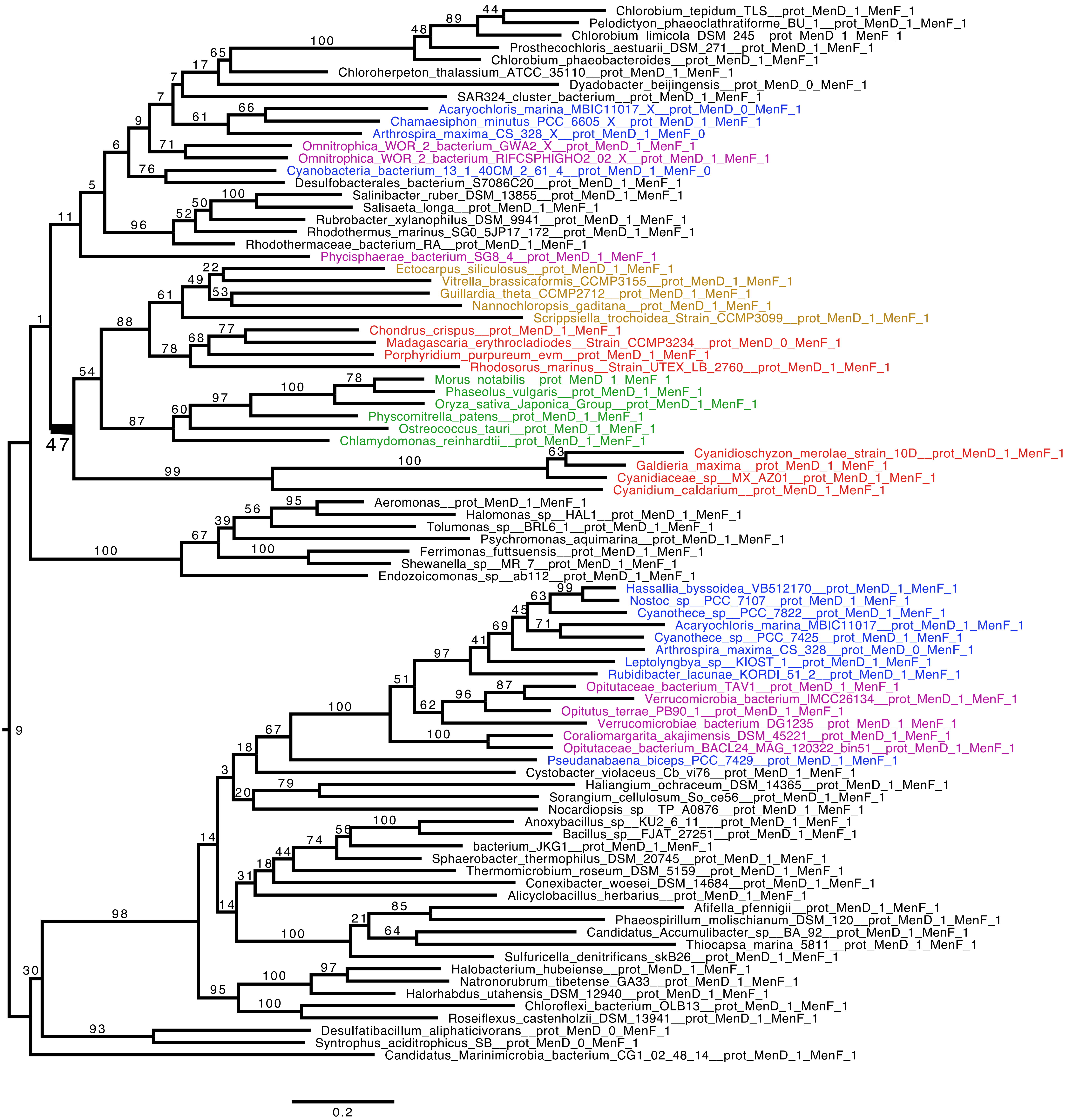

Supplementary figure S10. Concatenated phylogenetic analysis of MenFD without Chlamydiales from the same alignment than figure 3 and 4, as in supplementary figure 9. The tree displayed was performed with the same alignment than panel A but using IQ-TREE (Nguyen et al., 2015) under the C30 model (Le et al., 2008). Tree is manually rooted to easily compare with tree displayed in supplementary figure 9. The scale bar shows the inferred number of amino acid substitutions per site. The Rhodophyta are displayed in red, Chloroplastida are stained in green, algae bearing a plastid obtained by secondary or tertiary plastid endosymbiosis are shown in brown, all other taxa are displayed in black. Compare to Figure 4, when we remove Chlamydiales we could reinforce the monophyly of the Archaeplastida by increase of the bootstrap support for Archaeplastida (BV = 47%), however the statistical support of the nodes falls short of the required values to strongly support this conclusion.

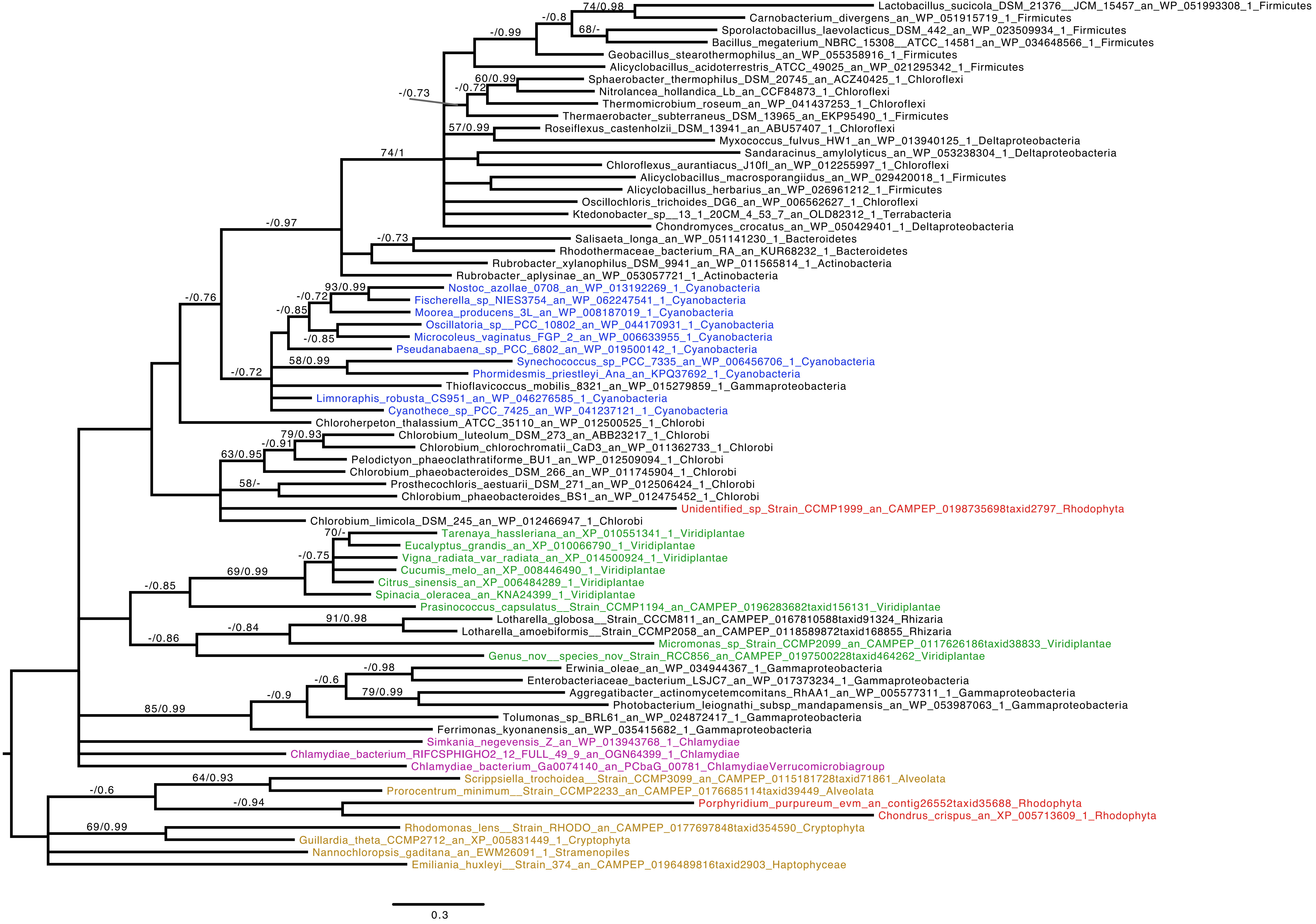

Supplementary figure S11. MenH phylogenetic tree with emphasis on Simkaniaceae and Archaeplastida phylogenetic analysis from the plastidial Cyanidiophytina Men cluster genes. The tree displayed is the consensus tree obtained with Phylobayes (Lartillot et al., 2009) under the CAT+GTR models (Lartillot and Philippe, 2004) with ML boostrap values obtained with the C10 (left) and Bayesian posterior probabilities (right) mapped onto the nodes. Bootstrap values (BV) >50 % are shown, while only posterior probabilities (BP) >0.6 are shown. The trees are midpoint rooted. Sequences are colored according to their taxonomic affiliation: Chlamydiae are in purple, Cyanobacteria in dark blue, Viridiplantae are in green, Glaucophyta are in light blue, Rhodophyta are in red and algae bearing a plastid obtained by secondary or tertiary plastid endosymbiosis are shown in brown, other organisms are in black.

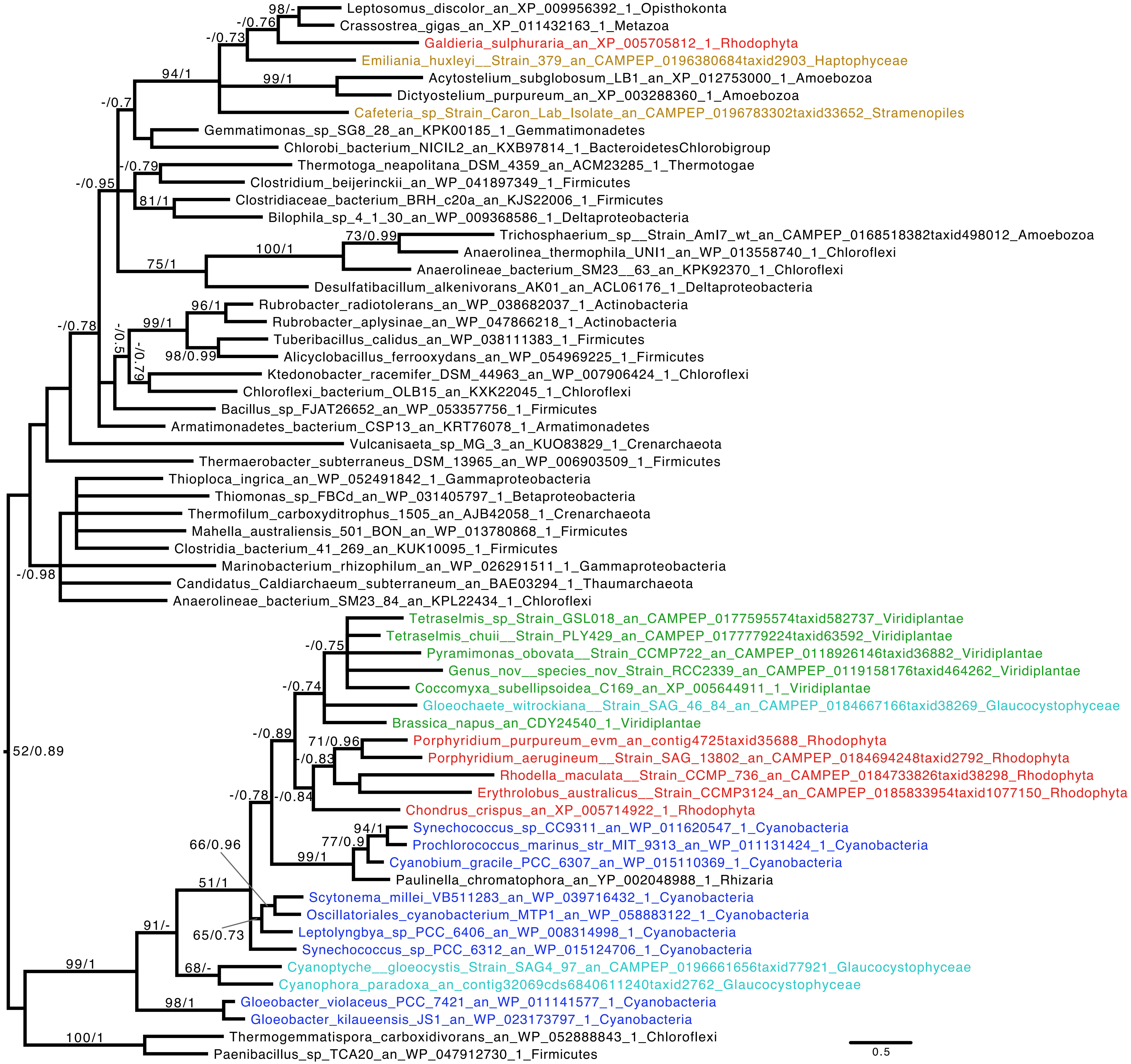

Supplementary figure S12. MenA phylogeny with emphasis on cyanobacteria, green algae and glaucophytes. Note that the apparent second MenA isoform in *G. sulphuraria* may be related to mitochondrial proteins involved in prenylation of other quinones. The tree was obtained as in supplementary figure 11.

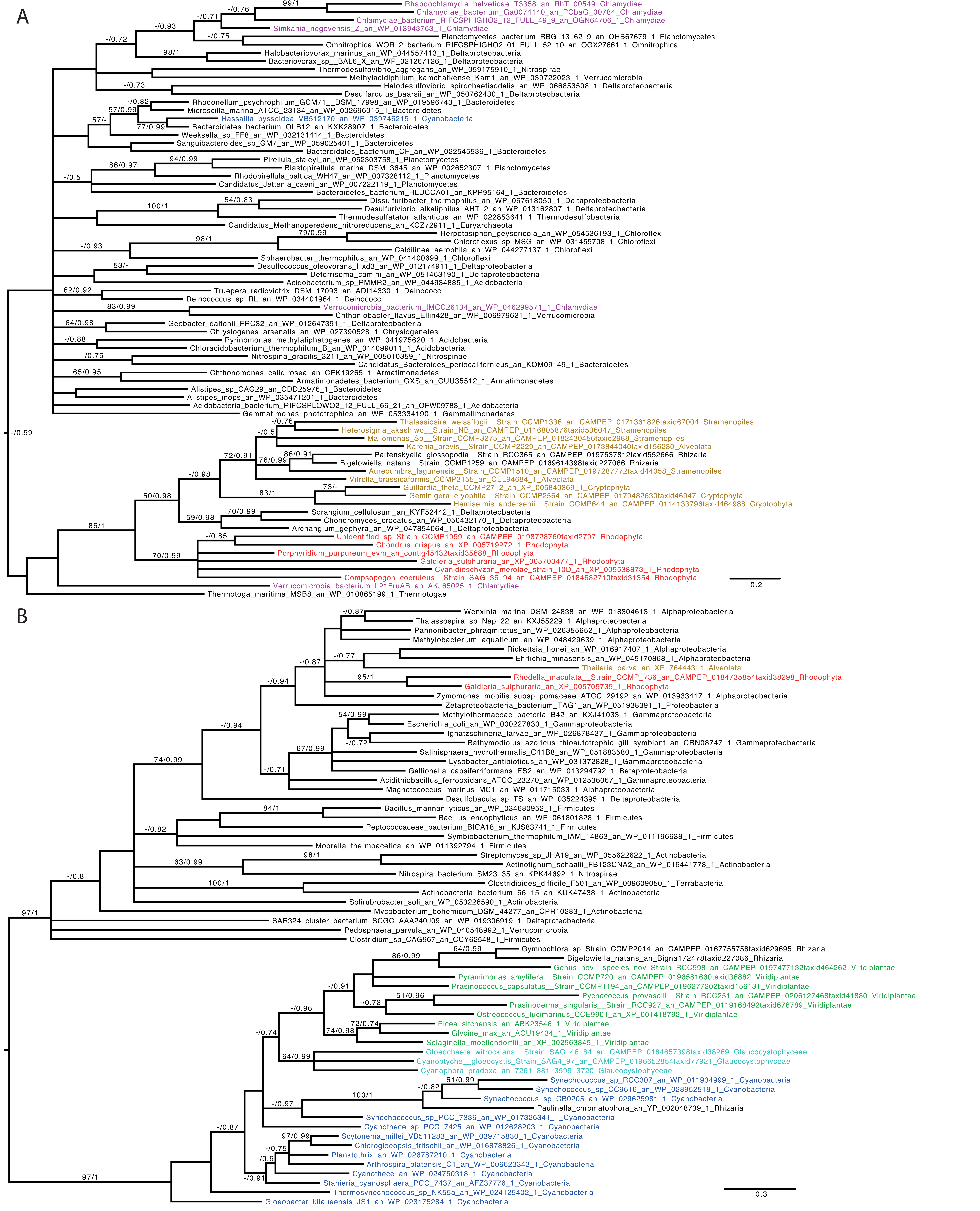

Supplementary figure S13. MenG phylogeny with emphasis on Rhodophyta and PVC group (A) or on Cyanobacteria and other Archaeplastida (B). The tree displayed is the consensus trees obtained with Phylobayes (Lartillot et al., 2009) under the CAT+GTR models (Lartillot and Philippe, 2004) with ML boostrap values obtained with the C20 model (left) and Bayesian posterior probabilities (right) mapped onto the nodes. Bootstrap values (BV) >50 % are shown, while only posterior probabilities (BP) >0.6 are shown. Sequences are colored according to their taxonomic affiliation as in supplementary figure 11.

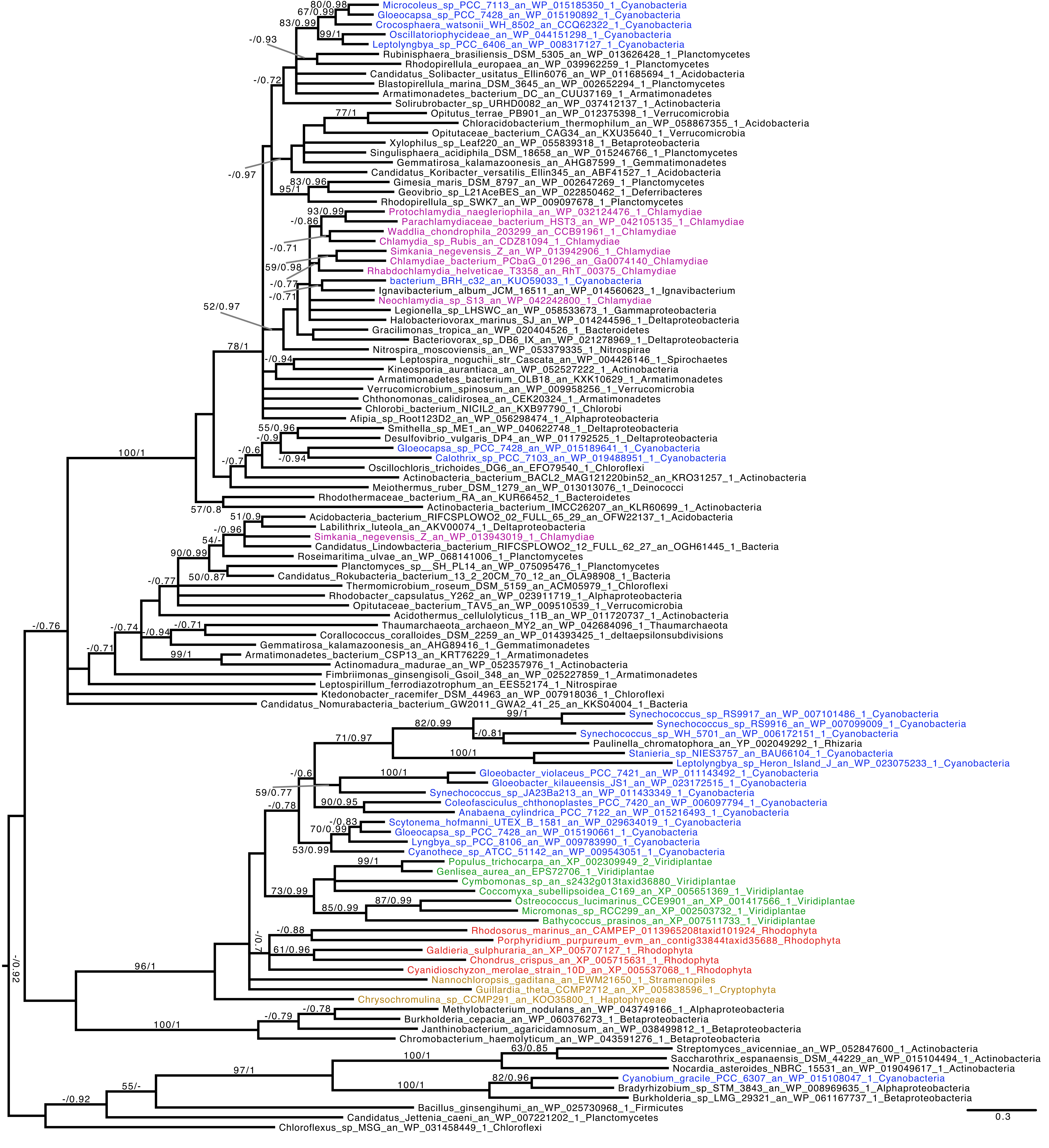

Supplementary figure S14. Ndc1 phylogenetic analysis. The tree was obtained and displayed as in supplementary figure 13.

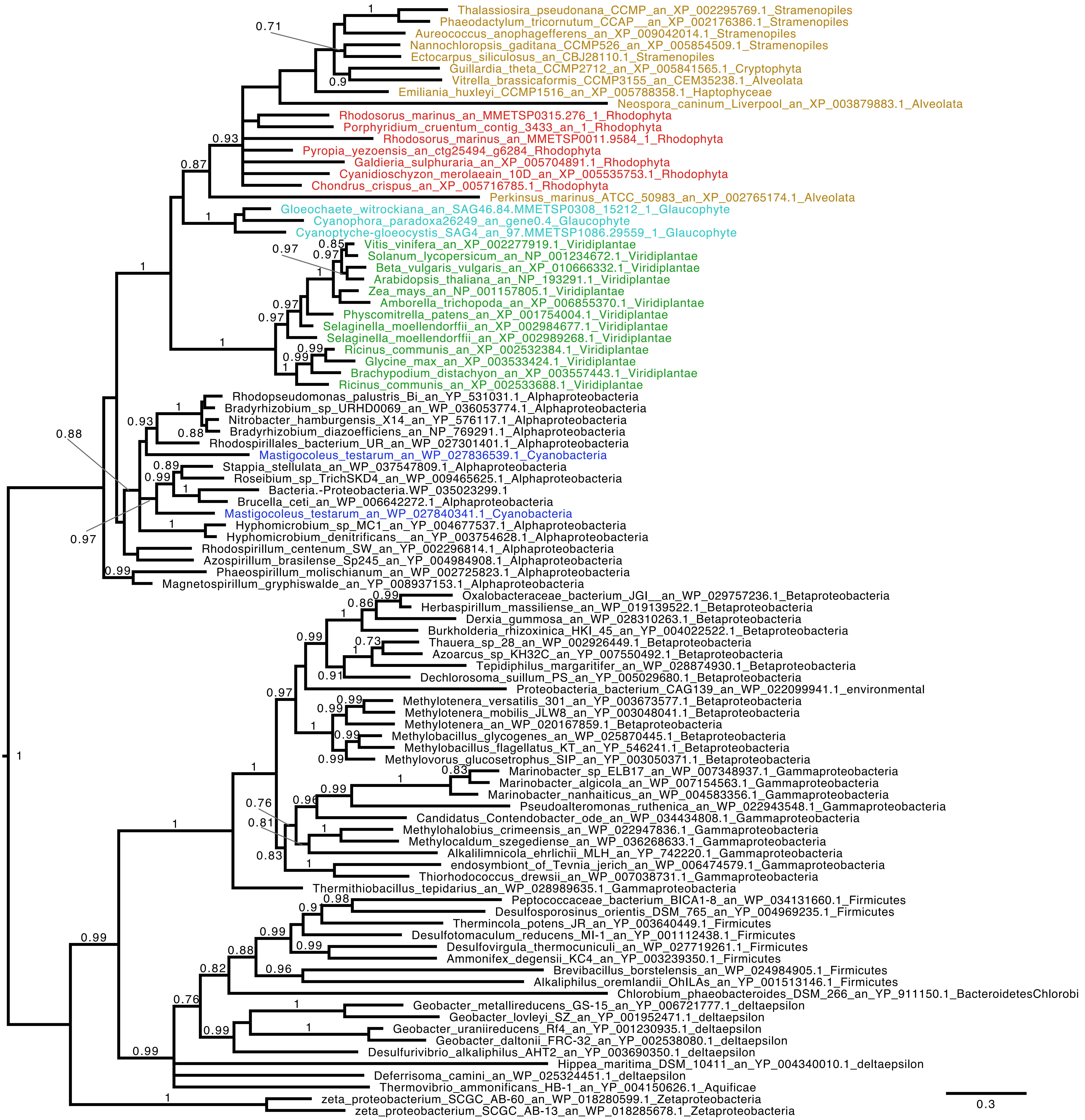

Supplementary figure S15. DOXP synthase (Dxs) phylogenetic analysis from the Isoprenoid MEP synthesis pathway. The trees shown are the consensus trees obtained with Phylobayes (Lartillot et al., 2009) under the CAT+GTR models (Lartillot and Philippe, 2004) and Bayesian posterior probabilities mapped onto the nodes, only posterior probabilities (pp) >0.7 are shown. The trees are midpoint rooted. Sequences are colored according to their taxonomic affiliation: Chlamydiae are in purple, Cyanobacteria in dark blue, other bacteria are in black, Viridiplantae are in green, Glaucophyta are in light blue Rhodophyta in red, and algae bearing a plastid obtained by secondary or tertiary plastid endosymbiosis are shown in brown.

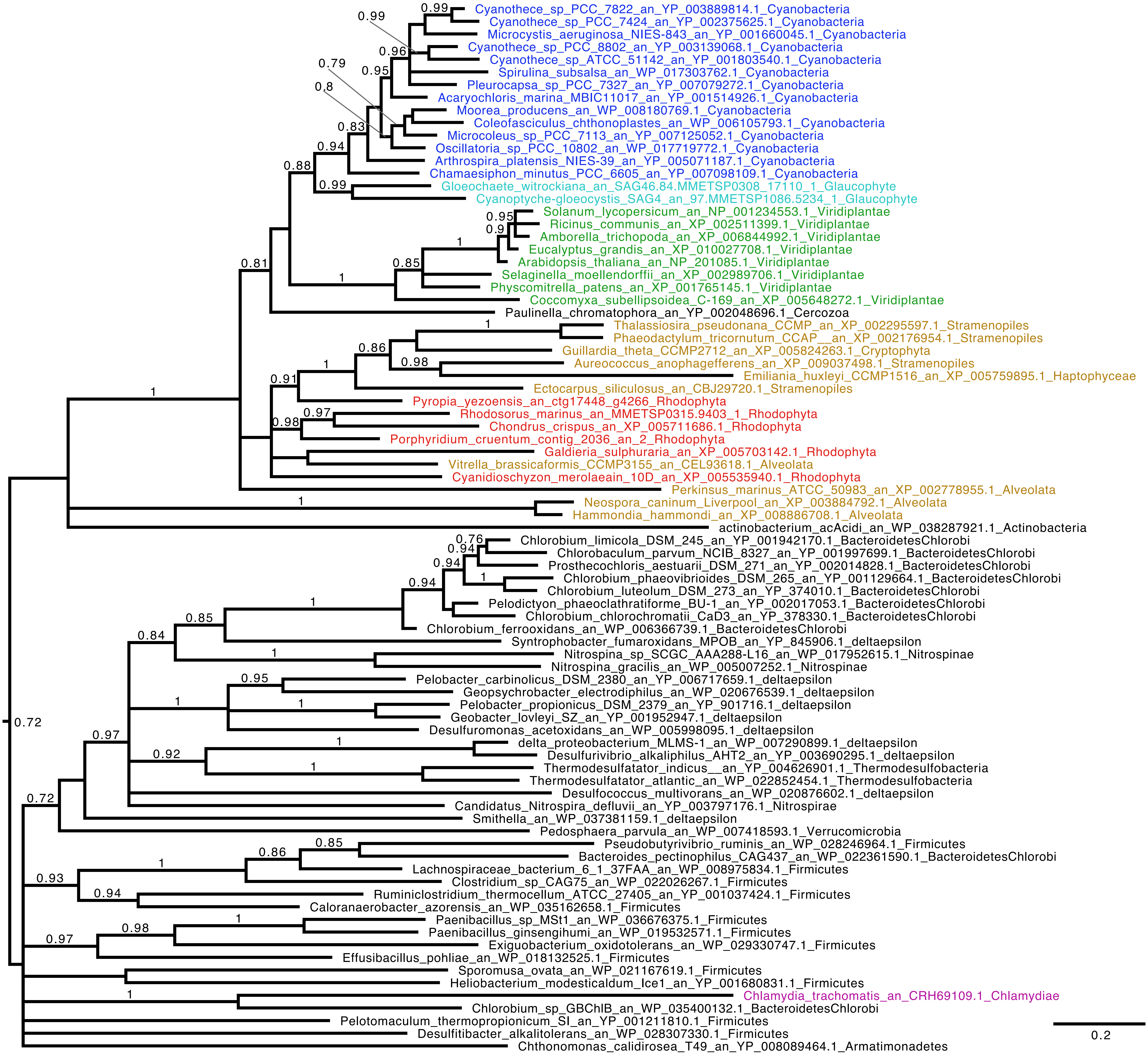

Supplementary figure S16. DOXP reductase (IspC) phylogenetic analysis from the Isoprenoid MEP synthesis pathway. The tree was obtained, colored and rooted as in figure 15.

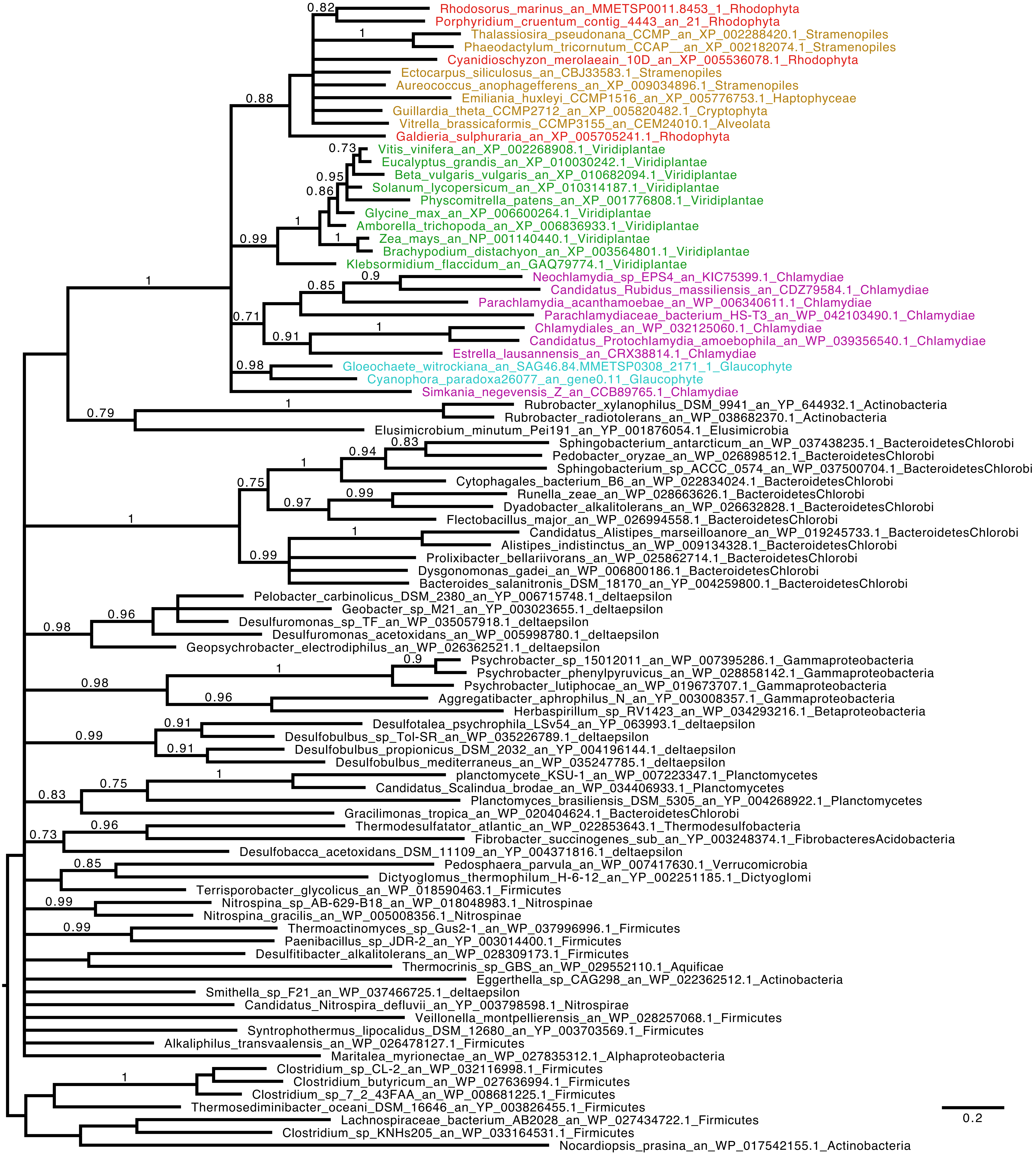

Supplementary figure S17. CDP ME synthase (IspD) phylogenetic analysis from the Isoprenoid MEP synthesis pathway. The tree was obtained, colored and rooted as in supplementary figure 15.

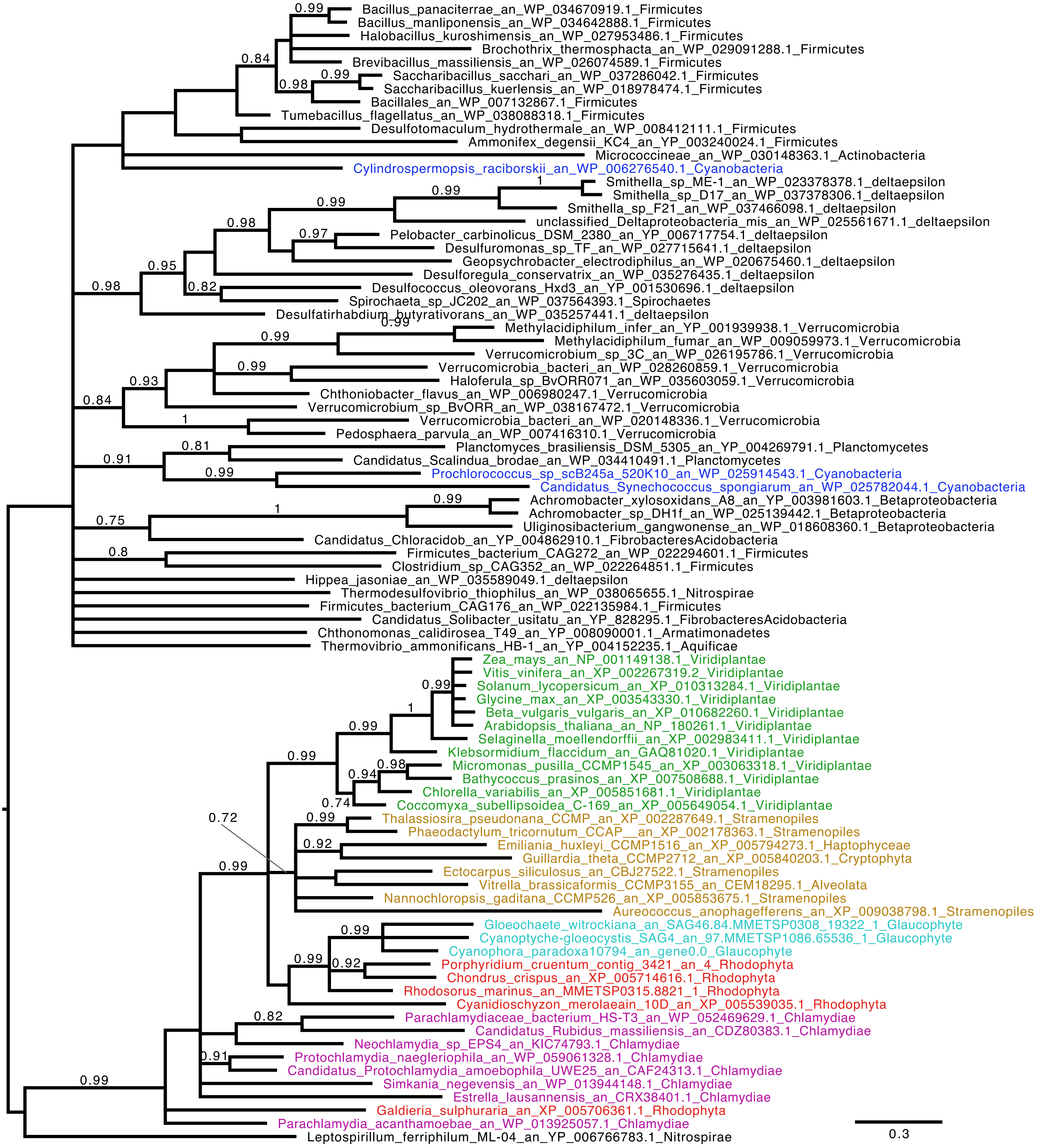

Supplementary figure S18. CDP ME Kinase (IspE) phylogenetic analysis from the Isoprenoid MEP synthesis pathway. The tree was obtained, colored and rooted as in supplementary figure 15.

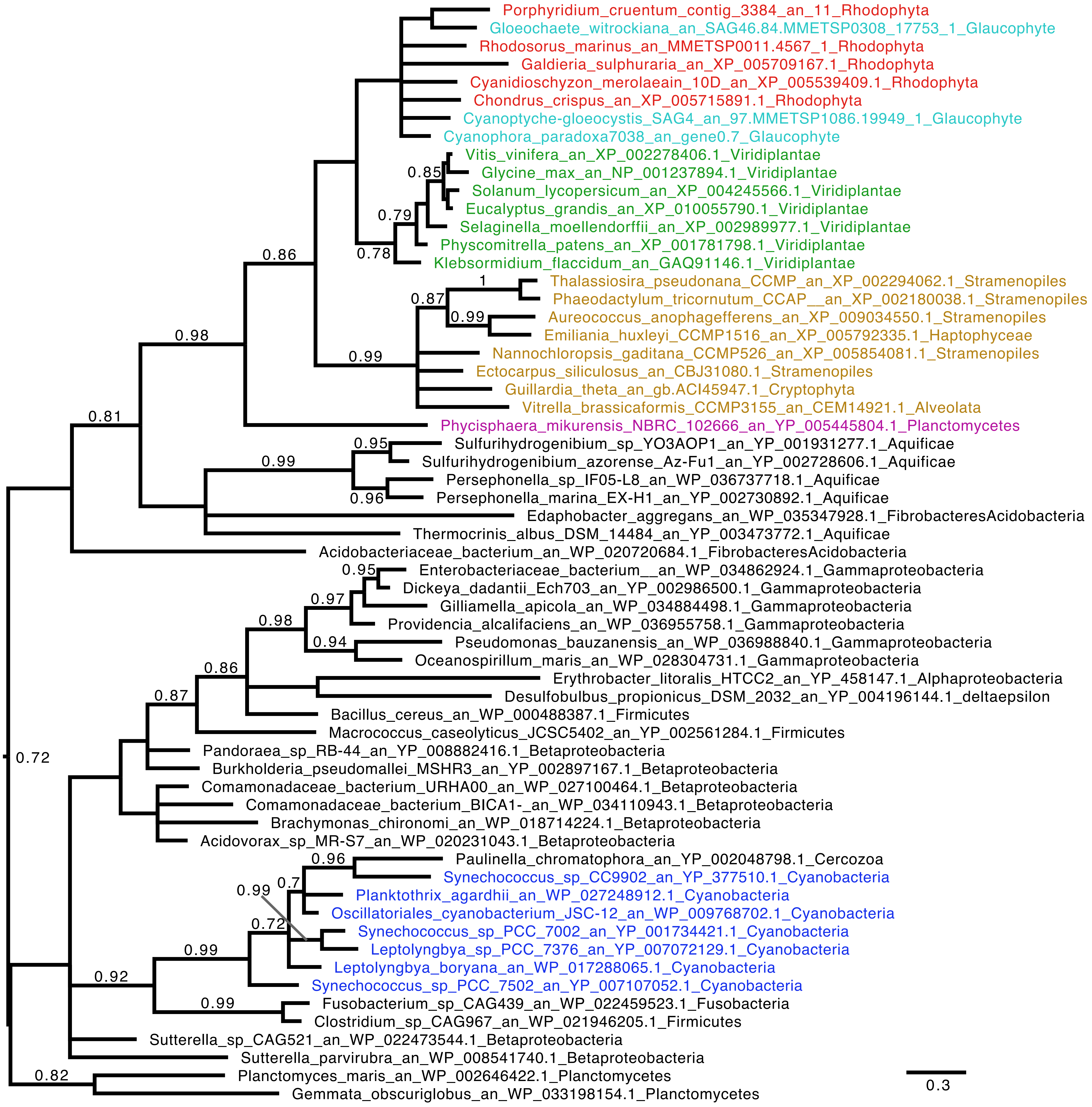

Supplementary figure S19. MEcPP synthase (IspF) phylogenetic analysis from the Isoprenoid MEP synthesis pathway. The tree was obtained, colored and rooted as described in supplementary figure 15.

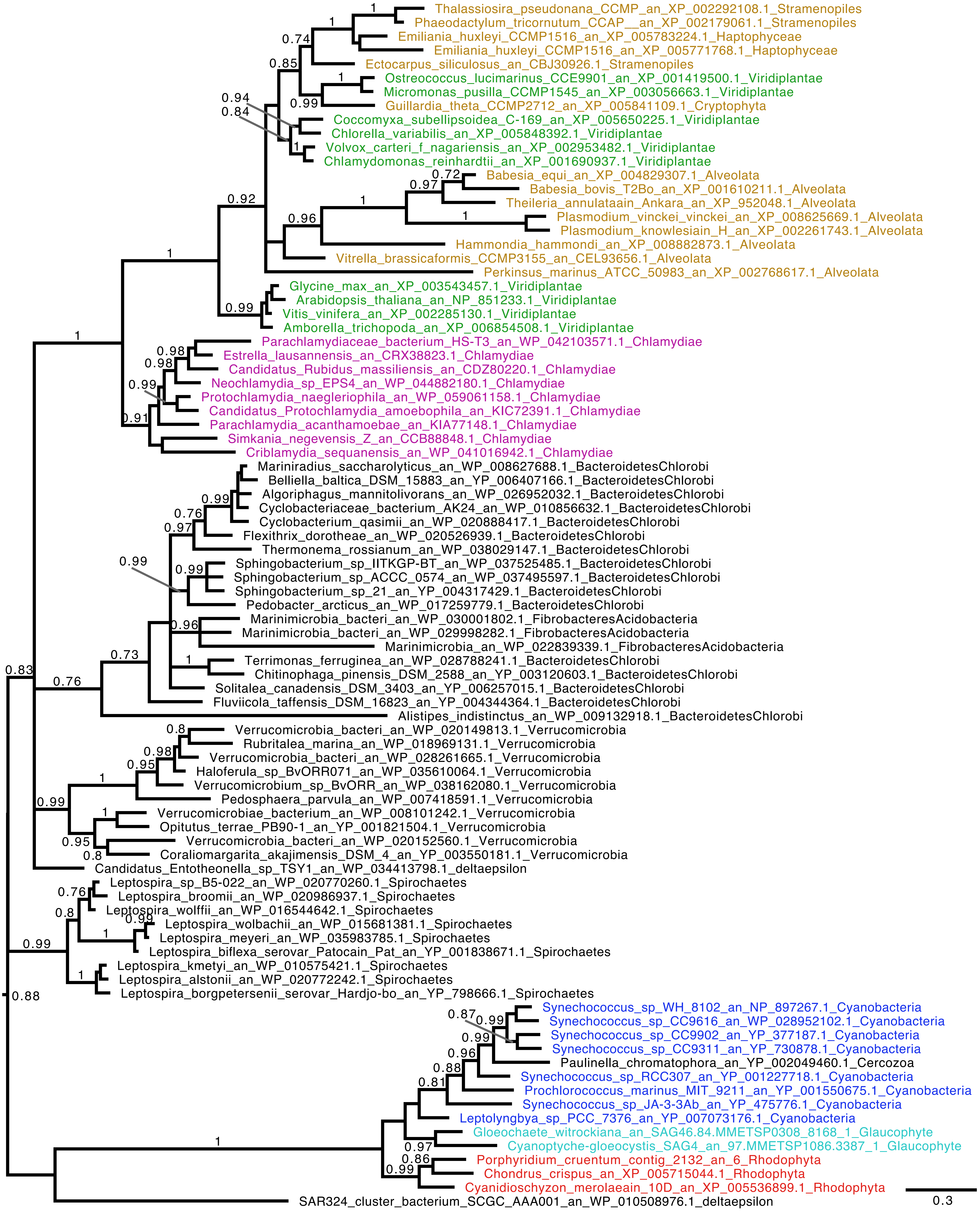

Supplementary figure S20. HMBPP synthase (IspG) phylogenetic analysis from the Isoprenoid MEP synthesis pathway. The tree was obtained, colored and rooted as in supplementary figure 15.

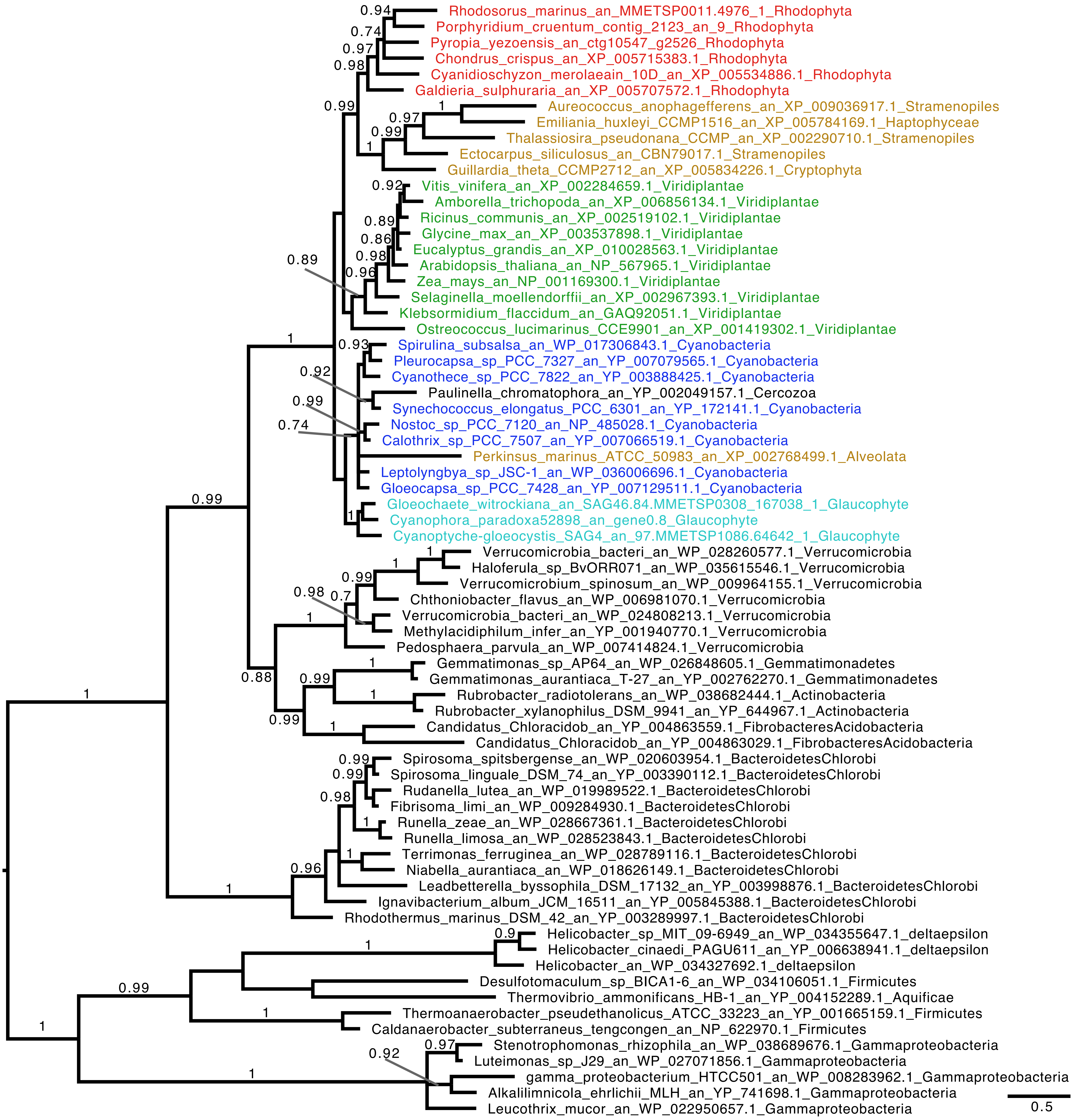

Supplementary figure S21. HMBPP reductase (IspH) phylogenetic analysis from the Isoprenoid MEP synthesis pathway. The tree was obtained, colored and rooted as described in supplementary figure 15.

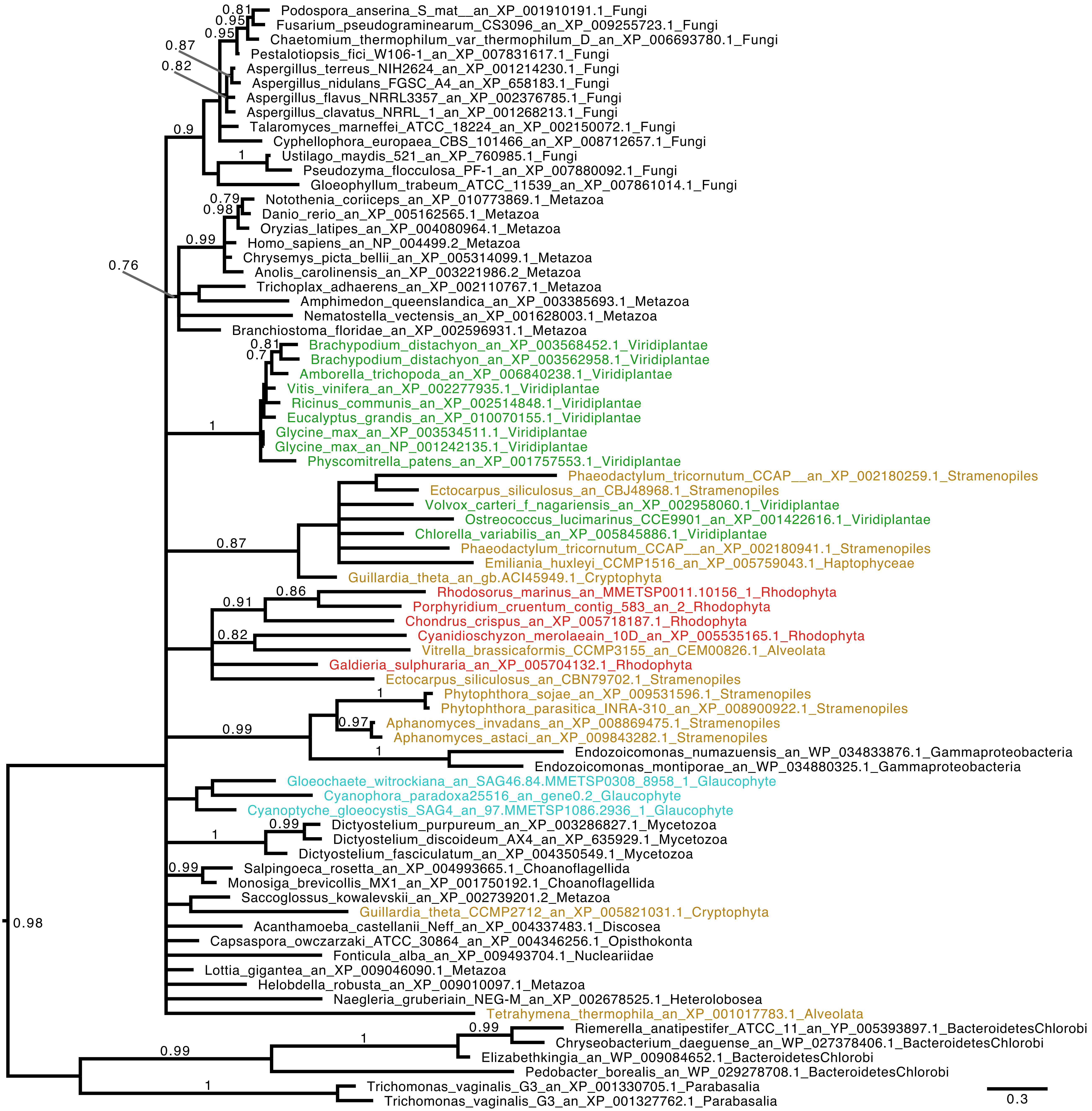

Supplementary figure S22. Isopentenyldiphosphate isomerase (IPP isomerase) phylogenetic analysis from the Isoprenoid MEP synthesis pathway. The tree was obtained, colored and rooted as described in supplementary figure 15.

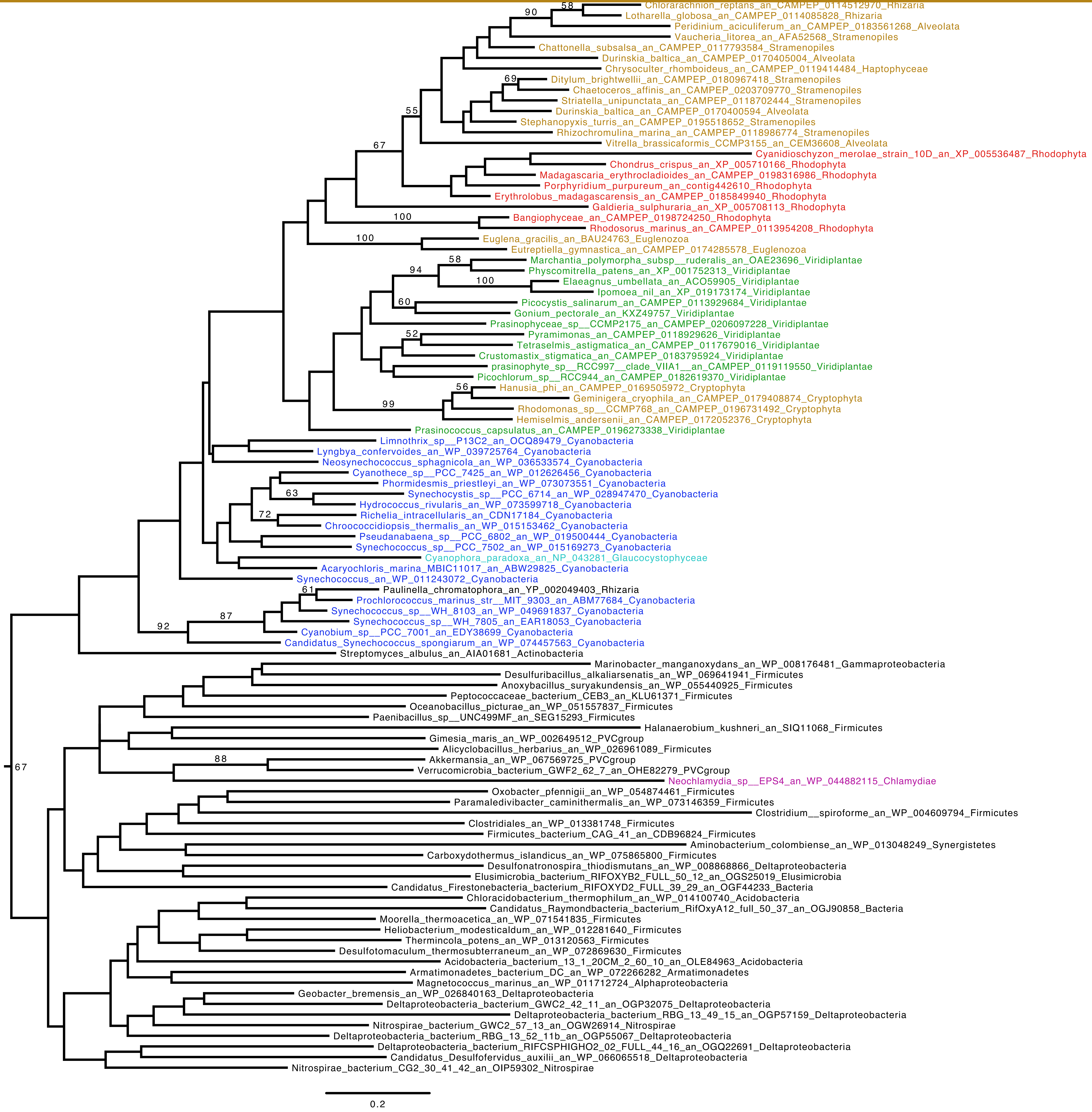

Supplementary figure S23. Geranyl geranyl diphosphate synthase (GGPS) phylogenetic tree from the Phytyl biosynthesis pathway. The tree shown was inferred by ML C20 analysis. Bootstrap values (BS) >50 % are shown. Sequences are colored according to their taxonomic affiliation: Chlamydiae are in purple, Cyanobacteria in dark blue, other bacteria are in black, Viridiplantae are in green, Rhodophyta are in red, and algae bearing a plastid obtained by secondary or tertiary plastid endosymbiosis are shown in brown, heterotrophic eukaryotes are displayed in grey. The scale bar shows the inferred number of amino acid substitutions per site.

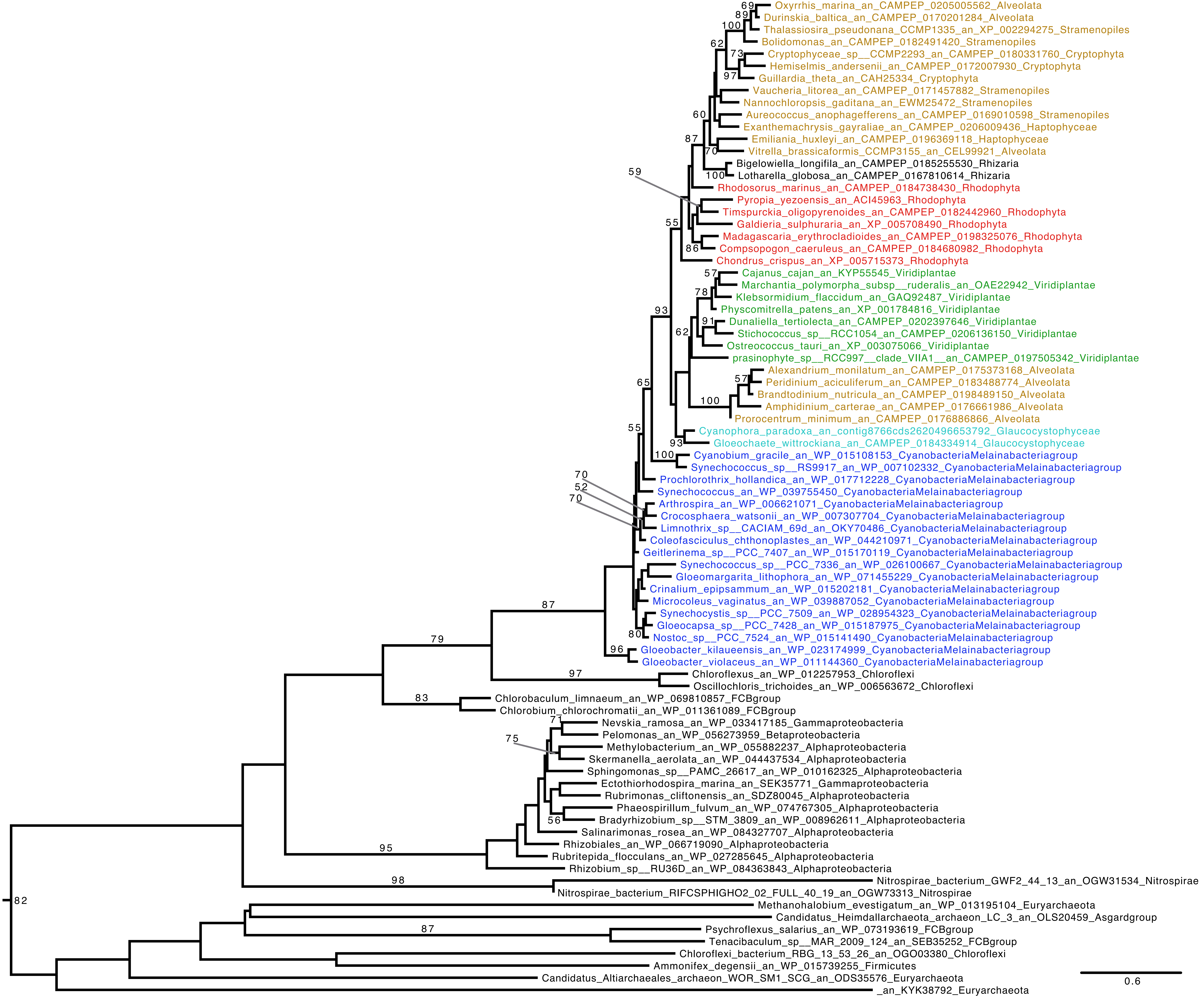

Supplementary figure S24. Geranyl geranyl diphosphate reductase (GGPR) phylogenetic tree from the Phytyl biosynthesis pathway. This tree was performed and is displayed as in supplementary figure 23.

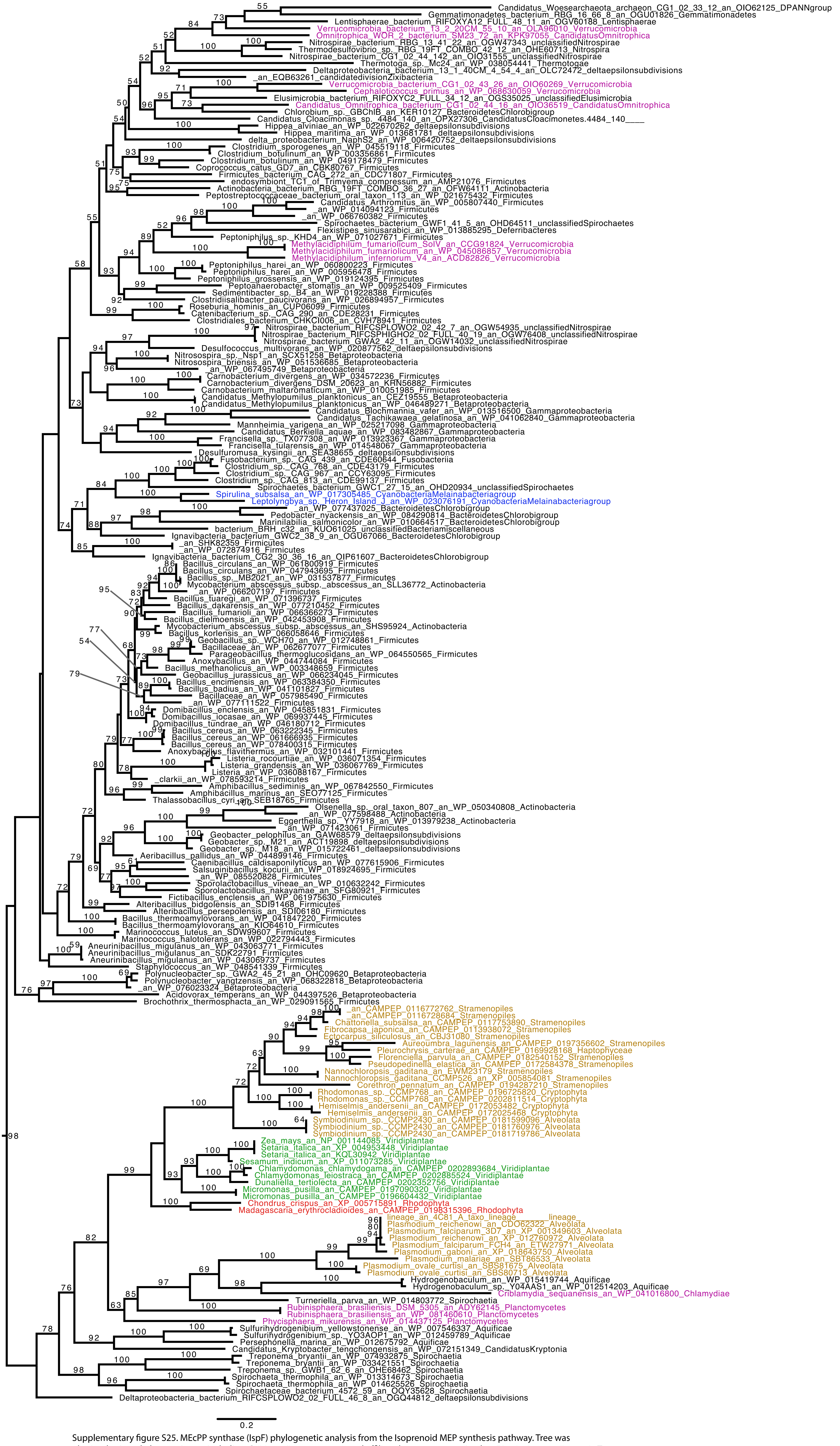

Supplementary figure S25. MEcPP synthase (IspF) phylogenetic analysis from the Isoprenoid MEP synthesis pathway. Tree was obtained using phylogenetic tree including *Criblamydia sequanensis* and affiliated sequences proximal to *Phycisphaera mikurensis*. To generate this tree the Criblamydia sequence was taken as a starting point for blast homology searches and clade selection. The tree is stained and rooted as described in supplementary figure 15.

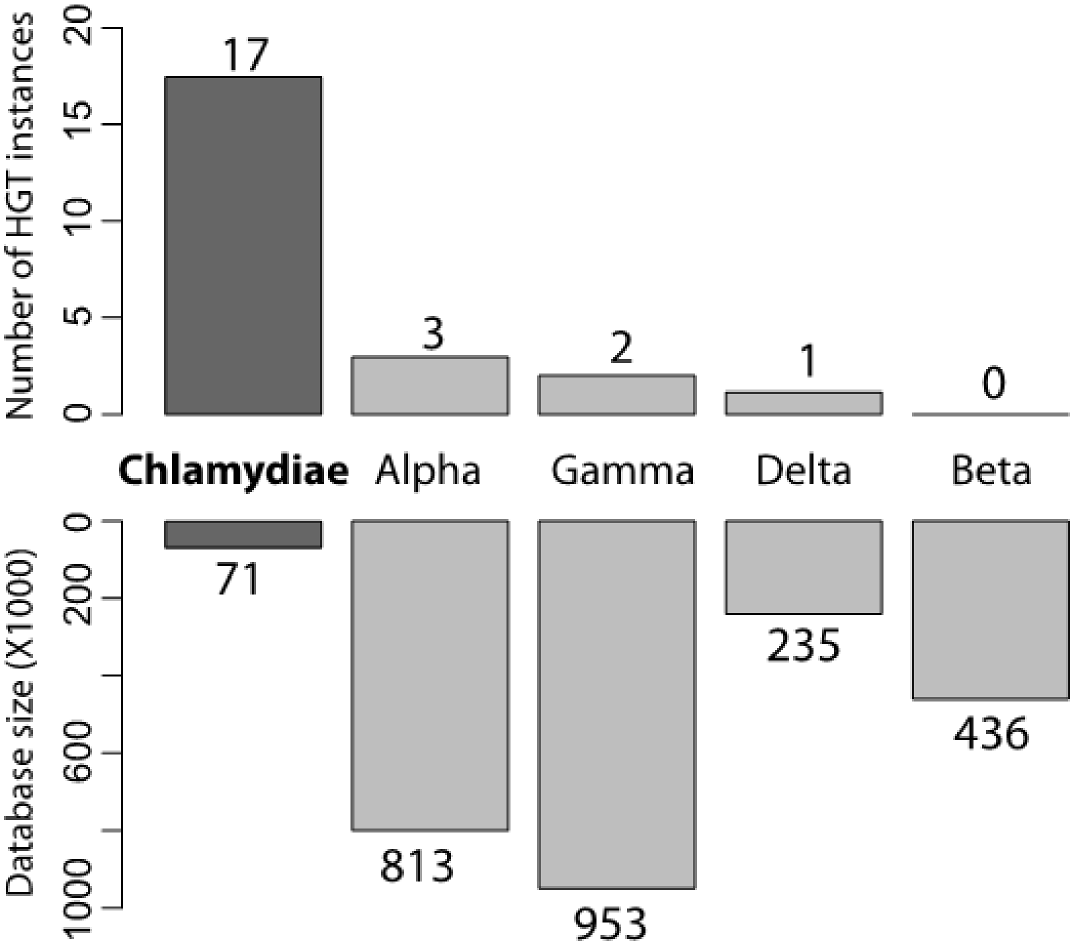

Supplementary figure S26. Contributions of Chlamydiae and Proteobacteria to the *A. thaliana* plastid proteome. The numbers of LGT from each donor source are shown in the upper panel with corresponding database sizes are shown in the lower panel. Chlamydia is shown in dark grey color and proteobacterial phyla in light grey color.

Table S1. List of chlamydial and proteobacterial derived LGTs found in *A. thaliana* plastid proteome data.

**No.**	**Chlamydiae**	**UFboot**	**Products**
1	AT1G32200	Exclusive	glycerol-3-phosphate chloroplastic
2	AT1G74960	100	beta-ketoacyl-ACP synthase
3	AT2G02500	100	2-c-methyl-d-erythritol 4-phosphate
4	AT2G25830	100	transcriptional regulatory protein
5	AT2G39140	99	Suppressor of var2 variegation
6	AT2G39930	100	isoamylase chloroplastic
7	AT3G02660	100	probable tyrosine--trna mitochondrial
8	AT3G03710	99	polyribonucleotide nucleotidyltransferase
9	AT3G56040	Exclusive	UDP-glucose pyrophosphorylase 3
10	AT4G17300	98	asparagine-trna synthetase
11	AT4G33680	96	ll-diaminopimelate chloroplastic
12	AT4G37270	100	cadmium zinc-transporting atpase
13	AT4G38020	100	tRNA/rRNA methyltransferase (SpoU) family protein
14	AT5G10910	96	methyltransferase-like protein 15
15	AT5G17990	100	anthranilate chloroplastic-like isoform x1
16	AT5G58330	99	lactate/malate dehydrogenase family protein
17	AT5G60600	98	hydroxy-2-methyl-2-(E)-butenyl 4-diphosphate (HMBPP) synthase (HDS)
	**Alphaproteobacteria**		
1	AT1G06130	98	glyoxalase 2-4
2	AT2G19940	99	oxidoreductases
3	AT5G08280	99	Hydroxymethylbilane synthase
	**Gammaproteobacteria**		
1	AT1G22410	96	Class-II DAHP synthetase family protein
2	AT5G03880	100	thioredoxin family protein
	**Deltaproteobacteria**		
1	AT1G15140	95	FAD/NAD(P)-binding oxidoreductase

Exclusive: Exclusive gene sharing among Chlamydiae, Plantae and other algal lineages associated with red algal secondary endosymbiosis. In other words, the gene is specific to these group of taxa.
